# Oxidative amidation of benzaldehyde using a quinone/DMSO system as the oxidizing agent†

**DOI:** 10.1039/c9ra02893e

**Published:** 2019-06-10

**Authors:** Itzel Mejía-Farfán, Manuel Solís-Hernández, Pedro Navarro-Santos, Claudia A. Contreras-Celedón, Carlos Jesus Cortés-García, Luis Chacón-García

**Affiliations:** Laboratorio de Diseño Molecular, Instituto de Investigaciones Químico Biológicas, Universidad Michoacana de San Nicolás de Hidalgo, Edif. B-1, Ciudad Universitaria Francisco J. Múgica, s/n Morelia 58030 Michoacán Mexico lchaco@umich.mx; CONACYT-Universidad Michoacana de San Nicolás de Hidalgo, Edif. B-1, Ciudad Universitaria Francisco J. Múgica, s/n Morelia 58030 Michoacán Mexico

## Abstract

An efficient transition-metal-based heterogeneous catalyst free procedure for obtaining the oxidative amidation of benzaldehyde using quinones as oxidizing agents in low molar proportions is described here. Pyrrolylquinones (PQ) proved to be more suitable than DDQ and 2,5-dimethylbenzoquinone to conduct the oxidation process. Although the solvent itself acted as the oxidant with low to moderate yields, PQ/DMSO provided an efficient system for carrying out the reaction under operational simplicity, mild reaction conditions, short reaction times and high yields of the desired product. The scope of the method was evaluated with substituted benzaldehydes and secondary amines. Theoretical foundations are given to explain the participation of quinones as an oxidizing agent in the reaction.

## Abstract 2

An efficient transition-metal-based heterogeneous catalyst free procedure for obtaining the oxidative amidation of benzaldehyde using quinones as oxidizing agents in low molar proportions is described here.

## Graphical abstract

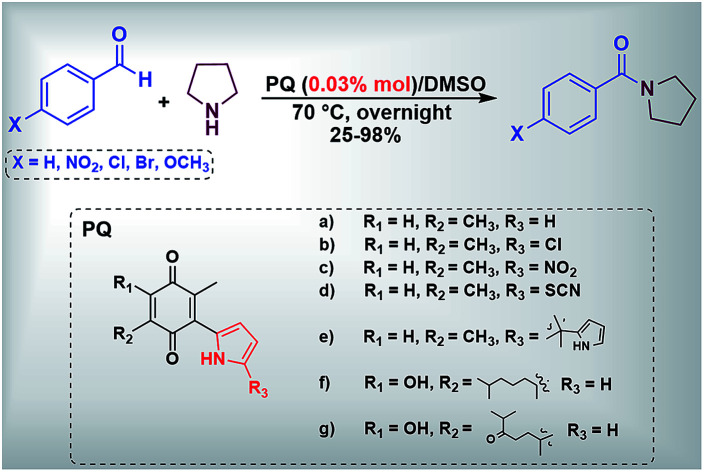

## Introduction

**Scheme 1:**
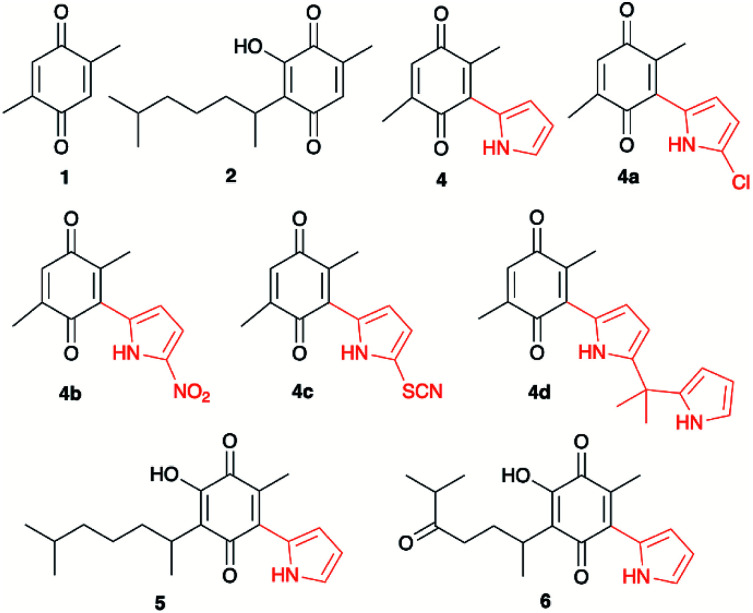
Quinones and pyrrolyl quinones used in this work.

Quinones, as for example 2,3-dichloro-5,6-dicyano-1,4-benzoquinone (DDQ), are used as oxidizing agents in organic synthesis.^1^ However, quinones are used as oxidants in organic synthesis as well as in biological systems. For example, 1,4-benzoquinone, that is, ubiquinone, couples NAD and electrons during oxidative phosphorylation in the electron transfer chain, toward the synthesis of ATP.^2^ Hydroquinone, a reduced form of quinone obtained as a product of the oxidation process, is oxidized to regenerate quinone, often in a simple step in the presence of oxygen. In prior studies, we reported the attainment of a new group of fluoride-recognizing quinone derivatives, the pyrrolyl quinones, from the natural product perezone and from 2,6-dimethyl-1,4-dibenzoquinone 1.^3^ The anion recognition capabilities of these compounds suggested that in addition to generating radicals, the pyrrolyl quinones were deficient electron species capable of participating in oxidative addition reactions. In this way, the pyrrolyl quinones could participate in oxidative amidation, in which an aldehyde is oxidized in the presence of an amine and an oxidizing agent to give the amide.^4^ The functional group amide is an important chemical linkage, essential for life because it forms the structural backbones of proteins.^5^ Amides form building blocks in organic synthetic chemistry and are prevalent in a variety of natural products, pharmaceuticals, agrochemicals, polymers, and materials.^6^ More than 25% of natural products and drug molecules possess an amide bond.^7^ Conventional amide synthesis methods involve coupling reactions between amines and carboxylic acids or acyl halides,^8^ anhydrides,^9^ esters,^10^ and acyl azides.^11^ Catalytic methods based on transition metals^12^ are considered to be environmentally unfavorable. Aldehydes have become important scaffolds for the synthesis of amides through oxidative amidation in recent years. Nakagawa *et al.* first reported the oxidative amidation of aldehydes with amines in the presence of ammonia and stoichiometric amounts of nickel peroxide as the oxidant.^4^ Recently, several groups reported photocatalytic methods based on phenazinium,^13^ Rose Bengal,^14^ BODIPY,^15^ quinolizinium compounds,^16^ and hemicyanine derivatives.^17^ This paper describes the use of pyrrolyl quinones (Scheme 1) as efficient oxidant in the oxidative amidation of benzaldehyde.

## Results and discussion

**Scheme 2:**
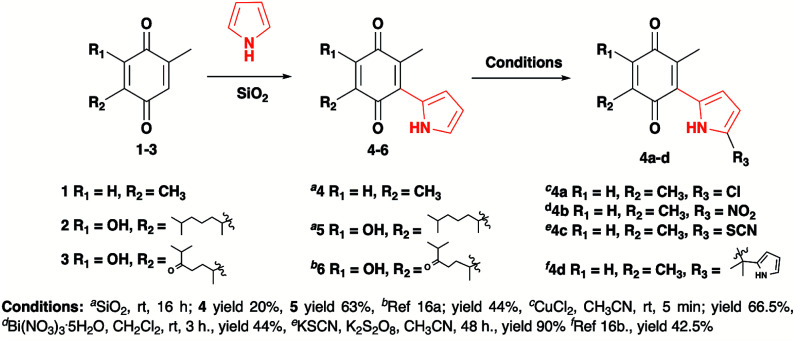
Synthesis of pyrrolyl quinones.

The pyrrolyl quinones were obtained from the corresponding quinones as summarized in Scheme 2.

Table 1 summarizes the results of the oxidative amidation between 4-nitrobenzaldehyde and pyrrolidine using the quinones, 1, 2, 4, 4a–4d, 5, 6, and DDQ. Both DDQ and 1,4-benzoquinone have been tested previously for the same purposes in comparison with other oxidizing without good results.^12*a*,17,18^ The quantity of oxidizing agent used in this work is very low. In a typical reaction, 0.02 mmol was reacted with 0.66 mmol aldehyde. The addition of more quinone did not increase the yield, and the use of less quinone reduced the yield (Entry 6, 7 and 8, Table 1).

**Table 1:** Oxidative amidation using quinones^a^

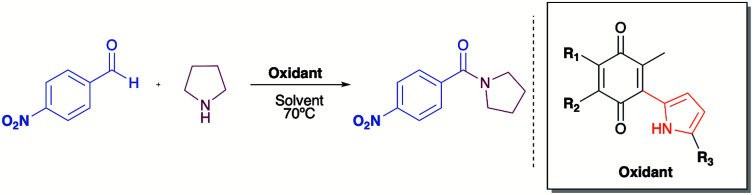								
Entry	Oxidant	R_1_	R_2_	R_3_	Yield^b^ (%)		DMSO	
					CH_3_CN	DMSO	Conversion (%)	Selectivity of product (%)
1	1	–H	–CH_3_	___	18	26	85	30.5
2	2	–OH	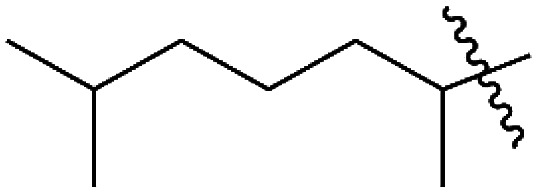	___	68	29	52	55.7
3	4	–H	–CH_3_	–H	23	45	81	55.5
4	4a	–H	–CH_3_	–Cl	30	54	80	67.5
5	4b	–H	–CH_3_	–NO_2_	55	64	80	80
6	4c	–H	–CH_3_	–SCN	85	98	100	98
7	c4c	–H	–CH_3_	–SCN	___	17	84	20
8	d4c	–H	–CH_3_	–SCN	___	41^e^	100	41
9	4d	–H	–CH_3_	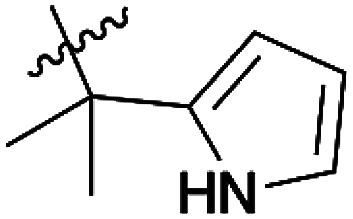	42	25	79	31.6
10	5	–OH	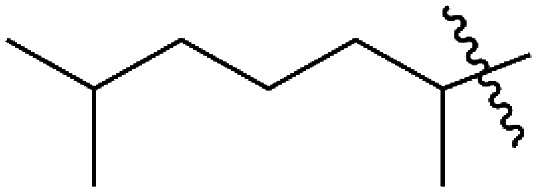	–H	73	33	66	50
11	6	–OH	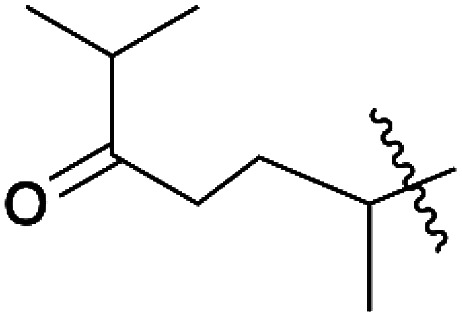	–H	58	27	78	34.6
12	DDQ	___	___	___	21	59	100	59

a Reagents and conditions: aldehyde (0.66 mmol), pyrrolidine (0.79 mmol), oxidant (0.02 mmol) solvent 2 ml (CH_3_CN or DMSO), 70 °C, 19 h.

b Isolated yields.

c Oxidant (0.01 mmol).

d Oxidant (0.04 mmol).

e Polar side products were found.

The reaction was carried out in acetonitrile and dimethylsulfoxide as both solvents are considered radical stabilizers.^19^ The change in solvent, from acetonitrile to dimethylsulfoxide, clearly altered the reaction efficiency, and the yield increased from 30–54% (input 4) to 85–98% (input 6) only by swapping acetonitrile with dimethylsulfoxide. For quinones 1, 4, 4a, 4b and 4c the advantages of DMSO were evident.

The pyrrolyl quinones generally provided better yields than the corresponding quinone parents 1 and 2. The best performance was obtained from thiocyanate 4c with 98% yield in very clean reaction (input 6).

The reaction yield obtained from 2.5-dimethyl-1.4-dibenzoquinone was very low (26%, entry 1). DDQ provided a 59% yield, but many by-products were produced, complicating the reaction purification processes.

As has been described in previous oxidative amidation work, the electroatractor groups substituted in the aromatic system favour the reaction by facilitating the nucleophilic attack of amine on carbonyl.^12*a*,13,14^ We explored the effects of the benzaldehyde substituents by conducting the reaction under the same conditions, but with various substituents at the benzaldehyde position 4 (–H, –NO_2_, –OCH_3_, –Cl, and –Br) using compound 4c as the oxidizing agent. The results are summarized in Table 2. For the halogen substituents, a change in solvent from acetonitrile to dimethylsulfoxide provided a marked yield increase, from 6 to 57% for 4-bromobenzaldehyde and from 8 to 71% for 4-chlorobenzaldehyde (Table 2).

**Table 2:** Oxidative amidation of different aldehydes^a^

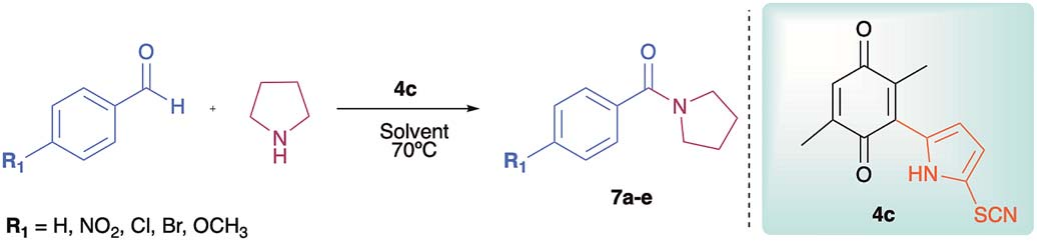			
Amide	R_1_	Yield (%)	
		DMSO	CH_3_CN
7a	–NO_2_	98	85
7b	–OCH_3_	86	56
7c	–H	64	35
7d	–Cl	71	8
7e	–Br	57	6

a Reagents and conditions: aldehyde (0.66 mmol), pyrrolidine (0.79 mmol), 4c (0.02 mmol), solvent (CH_3_CN or DMSO) 70 °C, 19 h. Isolated yields.

In order to evaluate the extent of oxidation to other secondary amines, the reaction was carried out with diethylamine, dibutylamine, morpholine and piperazine (Table 3).

**Table 3:** Reaction of 4-nitrobenzaldehyde with secondary amines^a^

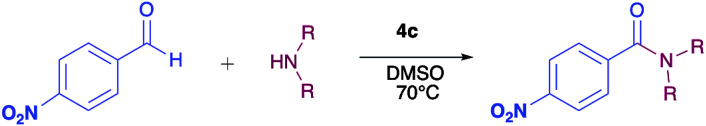			
Compound	Amine	Product	Yield (%)
8	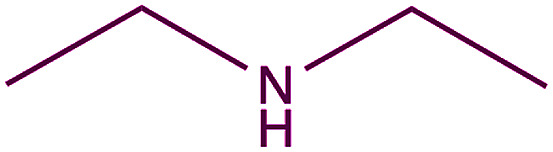	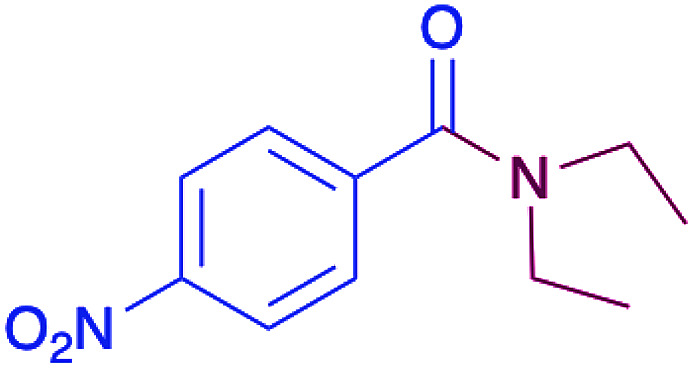	34
8a	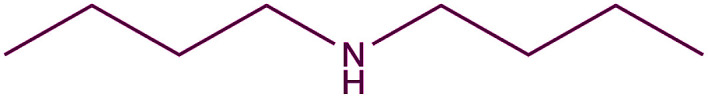	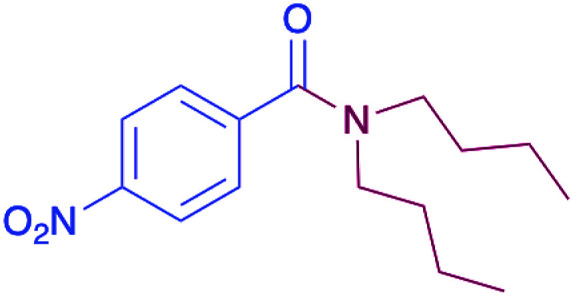	43
8b	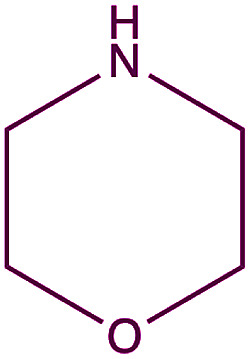	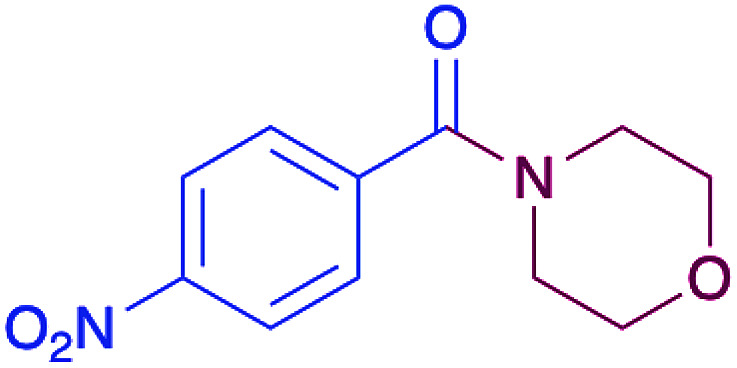	87
8c	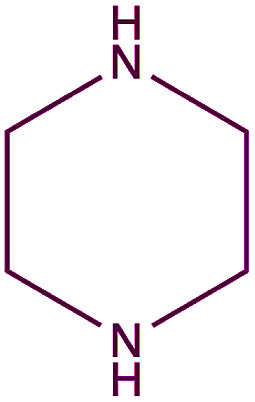	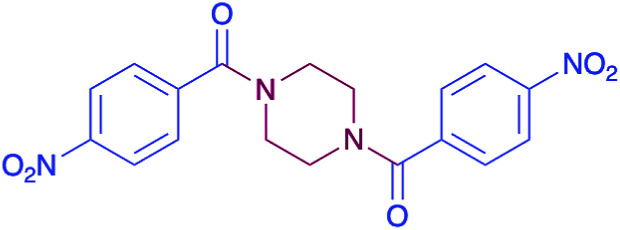	32

a Reagents and conditions: aldehyde (0.66 mmol), amine (1.2 eq), 4c (0.02 mmol), DMSO 2 ml, 70 °C, 19 h. Isolated yields.

To give theoretical insights about the participation of quinones as oxidizing agent, proper forms of the Fukui functions,^20^*f*(*r*), have been calculated to describe the local reactive sites of the pyrrolyl quinones. The reactivity is characterized through *f*(*r*), which describe the local changes occurring in the electron density *ρ*(*r*) due to changes in the number of electrons *N*.

The *f*(*r*)^0^ form of the Fukui functions was used as a stability descriptor pursuing zones within the pyrrolyl quinones that could stabilize a free radical. The *f*(*r*)^0^ descriptor indicated regions in the pyrrolyl quinones in which an unpaired electron could potentially be localized after redistribution of the initial electronic density.

The highest values of *f*(*r*)^0^ suggested that the oxygen atoms O_8_ and O_9_ of the quinones were the most favorable sites for stabilizing a free radical, with a subtle preference for O_8_ over O_9_. O_9_ participates in non-bonded interactions, whereas O_8_ can accept one electron to form a radical. Radical formation raises an interesting question: Do the pyrrolyl quinones accept or donate the electron? To address this question, we calculated the values of *f*(*r*)^+^ and *f*(*r*)^−^ of the Fukui functions in the open shell scheme (after radical formation). The value *f*(*r*)^+^ provides information about sites that stabilize incoming charges on the PQs. The value of *f*(*r*)^−^ gives information about the electron donor sites from which a charge may “exit” to stabilize the PQs in a subsequent step.

Table 4 indicates that the highest values of the Fukui function occurred at O_8_, particularly for *f*(*r*)^+^. Once the radical formed, O_8_ preferably accepted the incoming charge. It is important to note that *f*(*r*)^+^ increased in the presence of DMSO by up to 7.2%, in agreement with our proposed mechanism that the quinones promoted radical formation in the presence of DMSO with synergic effects.

**Table 4:** Condensed forms of *f*(*r*)^+^ and *f*(*r*)^−^, calculated after the radical formed in the PQs

Compound	Gas phase				DMSO			
	*f*(*r*)^+^		*f*(*r*)^−^		*f*(*r*)^+^		*f*(*r*)^−^	
	O_8_	O_9_	O_8_	O_9_	O_8_	O_9_	O_8_	O_9_
4	0.184	0.061	0.114	0.075	0.194	0.067	0.122	0.084
4a	0.181	0.069	0.129	0.078	0.194	0.066	0.120	0.083
4b	0.182	0.057	0.125	0.080	0.195	0.063	0.143	0.088
4c	0.184	0.061	0.120	0.078	0.196	0.067	0.138	0.087

Electron affinity (*A*), chemical potential (*μ*), hardness (*η*) and electrophilicity (*ω*) [in eV] of benzoquinones calculated in the presence of CH_3_CN and DMSO support the superiority of one solvent over another (see ESI† for more details).

Radicals are deficient species of electrons that can be stabilized or destabilized by inductive effects. However, according to the results, a direct relationship between the inductive capacity of the different substitutes of the pyrrolyl quinones and the yield of the amidation product is not appreciable.

The oxidation reaction was carried out with moderate to good yields under mild reaction conditions using very low amounts of quinone (0.02 mmol equivalents) compared with the use of peroxide, which required more than 1 molar equivalent. Interestingly 1.2 molar equivalents of amine were used, unlike other methodologies, which used 3 molar equivalents.^15,21^ The DMSO solvent provided a more efficient reaction than the acetonitrile solvent by increasing the yield and making the reaction cleaner. In previous work, the oxidative amidation of 2-oxoaldehydes^22^ was reported to use dimethyl sulfoxide as both the solvent and the oxidizing agent. In this case, the 2-oxoaldehydes possessed a neighboring carbonyl group that acted as an electron attractor and increased the reactivity of the aldehyde during the addition of the amine.

The increased reactivity facilitated the formation of an imine intermediary that presumably was responsible for the oxidation reaction, providing the corresponding amide and releasing dimethyl sulphide.^22,23^

The same treatment was applied to the aldehydes in this work, revealing that the quinone addition improved the reaction efficiency and cleanliness in a fraction of the reaction time, providing higher amide yields. The results are summarized in Table 5.

**Table 5:** Oxidative amidation in DMSO with and without quinone 4c

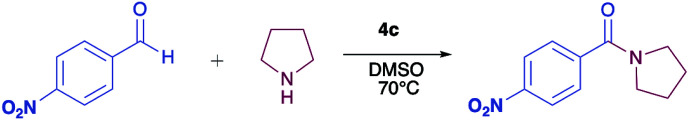				
Entry	Amide	R_1_	Yield (%)	
			aDMSO	bDMSO/4c
1	7a	–NO_2_	30	98
2	7b	–OCH_3_	21	86
3	7c	–H	16	64
4	7d	–Cl	19	71
5	7e	–Br	14	57

a Reagents and conditions: aldehyde (0.66 mmol), pyrrolidine (0.79 mmol), DMSO 2 ml, 70 °C, 19 h.

b Reagents and conditions: aldehyde (0.66 mmol), pyrrolidine (0.79 mmol), 4c (0.02 mmol), DMSO 2 ml, 70 °C, 19 h. Isolated yields.

DMSO radicals have been shown to be stable upon exposure to strong Brönsted–Lowry bases.^24^ Although pyrrolidine is not a strong base, quinone can promote radical formation in DMSO and *vice versa*. A mechanism involving cooperation between DMSO and quinone is, therefore, feasible.

**Scheme 3:**
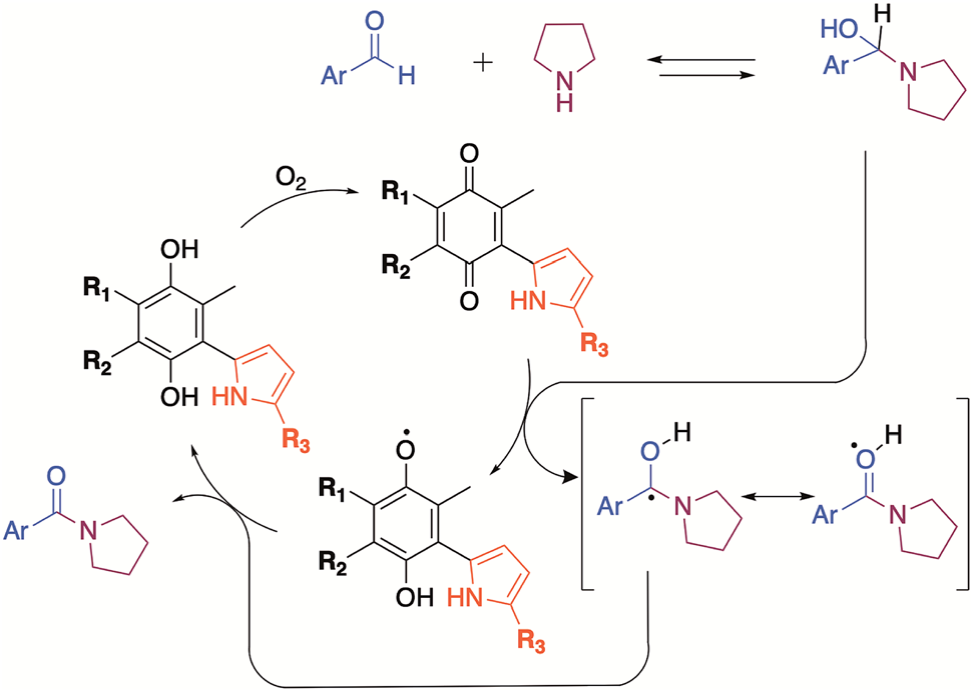
Mechanism proposed of oxidation with quinones.

The mechanism proposed here for the reaction is analogous to that proposed for a catalyst-free amidation assisted by an oxidizing agent such as peroxide (Scheme 3).

To verify the participation of oxygen in the proposed mechanism, experiments were carried out on acetonitrile and DMSO in open flask (presence of oxygen) and in argon atmosphere. The results summarized in Table 6 confirmed that oxygen is indeed necessary to carry out the reaction. The 30% yield obtained with DMSO in inert atmosphere is due to the fact that the solvent provides oxygen in the oxidation process by releasing dimethyl sulphide.^22^

**Table 6:** Oxidative amidation under inert atmosphere conditions and in the presence of oxygen^a^

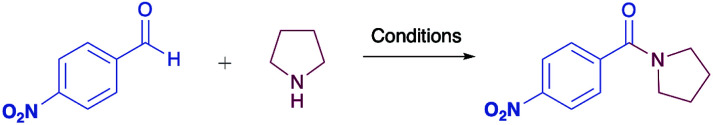		
Entry	Solvent	Yield (%)
b1	CH_3_CN	0
c2	CH_3_CN	28
b3	DMSO	30
c4	DMSO	29

a Reagents and conditions: aldehyde (0.66 mmol), pyrrolidine (0.79 mmol), 4c (0.02 mmol), solvent 2 ml (CH_3_CN or DMSO), 70 °C, 19 h.

b Open flask, without addition of 4c.

c Argon atmosphere, with addition of 4c.

## Conclusions

In conclusion, we have developed an efficient, metal-free oxidative amidation method under moderate reaction conditions with high yields which is, to our knowledge, the first report of this type of oxidation carried out by an organic oxidizing compound that is neither photoinductive nor peroxide. The solvents used in this work, acetonitrile and dimethylsulfoxide, are considered radical stabilizers that favor the formation of quinoid radicals generated by pyrrolylquinone facilitating the course of the reaction. We found that unlike acetonitrile, DMSO is able to carry out the oxidative amidation reaction of benzaldehyde in the absence of additive or catalyst although in low performance but interestingly, the reaction carried out in the presence of pyrrolylquinone/DMSO makes the reaction highly efficient.

## Conflicts of interest

There are no conflicts to declare.

## Supplementary Material

RA-009-C9RA02893E-s001
