# Supplementary material for: Oxidative amidation of benzaldehyde using a quinone/DMSO system as the oxidizing agent
Source: RSC Adv. 2019 Jun 10;9(32):18265–70. doi: 10.1039/c9ra02893e (PMC9064614; doi:10.1039/c9ra02893e)
Supplement: RA-009-C9RA02893E-s001 [file RA-009-C9RA02893E-s001.pdf]

Electronic Supplementary Material (ESI) for RSC Advances.
This journal is © The Royal Society of Chemistry 2019

Electronic Supporting Information

Oxidative amidation of benzaldehyde using a quinone/DMSO system as the oxidizing agent

Itzel Mejía-Farfán,[a] Manuel Solís-Hernández,[a] Pedro Navarro-Santos,[b] Claudia A. Contreras-Celedón,[a] Carlos Jesus Cortés-García[a] and Luis Chacón-García[a]*

[a]Laboratorio de Diseño Molecular, Instituto de Investigaciones Químico Biológicas Universidad Michoacana de San Nicolás de Hidalgo, Edif. B-1, Ciudad Universitaria, Francisco J. Múgica, s/n, Morelia 58030, Michoacán, Mexico

[b]CONACYT-Universidad Michoacana de San Nicolás de Hidalgo, Edif. B-1, Ciudad Universitaria, Francisco J. Múgica, s/n, Morelia 58030, Michoacán, Mexico

email: lchacon@umich.mx

Table of contents

**General information**	2
**Experimental Procedures**	2
**NMR Spectra**	5
**Theoretical calculations**	27
**References**	30

General information

All employed reagents were purchased from commercial sources and used without further purification. Nuclear magnetic resonance spectra were recorded on a Mercury 400 MHz. Chemical shifts were reported as δ values (PPM). Couplings constants *j* are reported in Hertz (Hz). Internal reference for NMR to TMS at 0.00 ppm for spectra obtained in $CDCl_3$), multiplicities are reports using the standard abbreviations, as follows: singlet (s), doublet (d), triplet (t), quartet (q), doublet of doublets (dd), broad signal (bs), doublet of triplets (dt), triplet of doublets (td), quartet of doublets (qd), multiplet (m), apparent triplet (at). NMR spectral were analyzed using MestReNova software (version 10.01-14719). HRMS spectral were acquired on a Bruker MicroTOF-II spectrometer. Melting points were determined on a Fisher-Johns melting point apparatus and are uncorrected. Reactions progress was monitored by thin layer chromatography (TLC) using silica gel 60 F254 from Merck and the spots were visualized under UV light at 245 or 365 nm. Column chromatography was performed using silica gel (230-400 mesh). Chemical names and drawings were obtained using ChemDraw Profesional (version 16.0.1.4 (61)).

Experimental Procedures

Synthesis of pyrrolyl quinones

2,5-dimethyl-3-(1*H*-pyrrol-2-yl)cyclohexa-2,5-diene-1,4-dione (4) The synthesis was carried out using a method described previously.[1] Flash Column chromatography (Hex:EtOAc 99:1) giving purple solid; yield 48 mg 20%, mp. 98-100 °C. **^{1}H NMR** (400 MHz, $CDCl_3$): δ 10.70 (s, 1H), 7.04 (td, *J* = 2.8, 1.3 Hz, 1H), 6.69 (ddd, *J* = 4.0, 2.5, 1.3 Hz, 1H), 6.60 (q, *J* = 1.5 Hz, 1H), 6.36 (dt, *J* = 3.9, 2.6 Hz, 1H), 2.28 (s, 3H), 2.05 (d, J = 1.5 Hz, 3H). **^{13}C NMR** (101 MHz, $CDCl_3$): δ 190.76, 187.04, 144.68, 134.88, 133.52, 131.71, 125.32, 122.25, 117.25, 109.91, 15.88, 14.75. **HRMS** (ESI$^+$): m/z: Calcd. for $C_{12}H_{11}NO_2$: calc. 201.0790; Found: 201.0783.

3-(5-chloro-1*H*-pyrrol-2-yl)-2,5-dimethylcyclohexa-2,5-diene-1,4-dione (4a). The synthesis was carried out using a method described in the literature with some modifications.[2] To a solution of 2,5-dimethyl-3-(1*H*-pyrrol-2-yl)cyclohexa-2,5-diene-1,4-dione. (100 mg, 0.496 mmol,) dissolved in CH_3CN (2.5 ml) was added $CuCl_2$ (33.4mg, 0.248 mmol). The mixture was stirred for 5 min. The solvent was removed under vacuum. The reaction crude was purified by flash column chromatography with Hexane:EtOAc 9:1 (v/v) giving purple solid; yield 78 mg, 66.5%, mp 103-105 °C. **^{1}H NMR** (400 MHz, $CDCl_3$): δ 10.70 (s, 1H), 6.63 (td, *J* = 4.1, 3.6, 2.2 Hz, 2H), 6.21 (dd, *J* = 4.0, 2.7 Hz, 1H), 2.27 (s, 3H), 2.07 (d, *J* = 1.6 Hz, 3H). **^{13}C NMR** (101 MHz, $CDCl_3$): δ199.65, 186.79, 144.75, 135.12, 133.74, 133.72, 130.85, 130.74, 124.61, 119.87, 118.23, 108.25, 15.91, 14.75. **HRMS**. (ESI$^+$): m/z: Calcd. for $C_{12}H_{11}NClO_2$: calc. 236.0478; Found: 236.0477.

2,5-dimethyl-3-(5-nitro-1*H*-pyrrol-2-yl)cyclohexa-2,5-diene-1,4-dione (4b). To a solution of 2,5-dimethyl-3-(1*H*-pyrrol-2-yl)cyclohexa-2,5-diene-1,4-dione. (30 mg, 0.149 mmol,) dissolved in CH_2Cl_2 (2.5 ml) was added $Bi(NO_3)_3 \cdot 5H_2O$ (36.15 mg, 0.0745 mmol,). The mixture was stirred for 3 h. The solvent was removed under vacuum. The residue was purified by flash column chromatography (Hex:EtOAc 7:3) giving yellow solid; yield 16 mg, 44%. mp. 116-118 °C. **^{1}H NMR** (400 MHz, $CDCl_3$): δ 11.33 (s, 1H), 7.20 – 7.13 (m, 1H), 6.73 (q, *J* = 1.6 Hz, 1H), 6.64 (dd, *J* = 4.4, 1.7 Hz, 1H), 2.33 (s, 3H), 2.13 (d, *J* = 1.6 Hz, 3H). **^{13}C NMR** (101 MHz, $CDCl_3$): δ 188.46, 186.23, 145.63, 141.81, 138.89, 13 3.67, 130.22, 128.49, 116.58, 110.37, 15.96, 14.75. **HRMS** (ESI$^+$): m/z: Calcd. for $C_{12}H_{10}N_2NaO_4$: calc. 269.0533; Found: 269.0514.

2,5-dimethyl-3-(5-thiocyanato-1*H*-pyrrol-2-yl)cyclohexa-2,5-diene-1,4-dione (4c) To a solution of 2,5-dimethyl-3-(1*H*-pyrrol-2-yl)cyclohexa-2,5-diene-1,4-dione. (100 mg, 0.496 mmol,) dissolved in CH_3CN (4 ml) was added KSCN (96.69 mg, 0.995 mmol) and $K_2S_2O_8$ (403 mg, 1.498 mmol). After 24 h was added KSCN (96.69 mg, 0.995 mmol). The solvent was removed under vacuum.[3] The residue was purified by flash column chromatography (Hex:EtOAc 4:1) giving red solid; yield 115mg 90% mp 123-125 °C. **^{1}H NMR** (400 MHz, $CDCl_3$): δ 10.84 (s, 1H), 6.73 (dd, *J* = 4.0, 2.7 Hz, 1H), 6.68 (q, *J* = 1.6 Hz, 1H), 6.65 (dd, *J* = 4.1, 2.6 Hz, 1H), 2.29 (s, 3H), 2.10 (d, *J* = 1.6 Hz, 3H).**^{13}C NMR** (101 MHz, $CDCl_3$): δ 189.56, 186.64, 145.24, 138.68, 133.61, 130.70, 130.39, 119.69, 117.81, 109.43, 107.81 **HRMS** (ESI$^+$): m/z: Calcd. for $C_{13}H_{10}N_2NaO_2S$: calc. 281.0355; Found: 281.0327.

3-(5-(2-(1*H*-pyrrol-2-yl)propan-2-yl)-1*H*-pyrrol-2-yl)-2,5-dimethylcyclohexa-2,5-diene-1,4-dione (4d). The synthesis was carried out using a method described previously.[4]

2-hydroxy-6-methyl-3-(6-methyl-5-oxoheptan-2-yl)-5-(1*H*-pyrrol-2-yl)cyclohexa-2,5-diene-1,4-dione (5). The synthesis was carried out using a method described previously.[1] Flash Column chromatography (Hex:EtOAc 99:1) giving purple solid; yield 79 mg 63%, mp. 53-55 °C. **^{1}H NMR** (400 MHz, $CDCl_3$): δ 7.11 (dt, *J* = 4.1, 2.1 Hz, 1H), 6.79 (ddd, *J* = 3.9, 2.3, 1.0 Hz, 1H), 6.42 – 6.33 (m, 1H), 3.06 (dp, *J* = 8.6, 7.0 Hz, 1H), 2.31 (s, 3H), 1.78 – 1.69 (m, 1H), 1.50 (dq, *J* = 13.2, 6.6 Hz, 2H), 1.22 (d, *J* = 7.1 Hz, 4H), 1.16 (ddq, *J* = 8.4, 4.6, 2.2 Hz, 3H), 0.83 (dd, *J* = 6.6, 4.1 Hz, 6H). **^{13}C NMR** (101 MHz, $CDCl_3$): δ. 190.89, 183.22, 150.99, 133.36, 129.00, 126.40, 123.89, 119.17, 110.49, 29.79, 27.82, 25.93, 22.54, 18.29, 14.44. **HRMS** (ESI$^+$): m/z: Calcd. for $C_{12}H_{11}NO_2$: calc.338.1727; Found: 338.1726.

2-hydroxy-6-methyl-3-(6-methylheptan-2-yl)-5-(1*H*-pyrrol-2-yl)cyclohexa-2,5-diene-1,4-dione (6). The synthesis was carried out using a method described previously.[1]

General Procedure for Oxidative Amidation.

A mixture of aldehyde (100 mg, 0.66 mmol), secondary amine (1.2 eq 0.79 mmol), quinone (0.02 mmol), in DMSO (2 mL) was stirred at 70 ºC for 19 h. in absence of light. The reaction mixture was concentrated in vacuo. The residue was purified by column chromatography on silica gel using hexane/EtOAc as eluent to obtain tertiary amide products.

(4-nitrophenyl)(pyrrolidin-1-yl)methanone (7a)
N-(4-Nitrobenzoyl)pyrrolidine,[5] Column chromatography (Hexane:EtOAc 80:20) giving yellow solid. Yield 143 mg, 98%. **^{1}H NMR** (400 MHz, $CDCl_3$); δ 8.27 (d, *J* = 8.7 Hz, 2H), 7.68 (d, *J* = 8.7 Hz, 2H), 3.67 (t, *J* = 6.9 Hz, 2H), 3.39 (t, *J* = 6.6 Hz, 2H), 2.04 – 1.97 (m, 2H), 1.96 – 1.89 (m, 2H).

(**4-methoxyphenyl)(pyrrolidin-1-yl)methanone (7b)**
Column chromatography (Hexane:EtOAc 80:20) giving a colorless oil 129 mg, 86%. ^{1}H NMR: 7.52 (d, *J* = 8.7 Hz, 2H), 6.89 (d, *J* = 8.7 Hz, 2H), 3.82 (s, 3H), 3.63 (t, *J* = 7.0 Hz, 2H), 3.47 (t, *J* = 6.5 Hz, 2H), 1.98-1.83 (m, 4H).

phenyl(pyrrolidin-1-yl)methanone (7c)
Column chromatography (Hexane:EtOAc 80:20) giving a Yellow oil 117 mg, 64%. ^{1}H NMR

(400 MHz, $CDCl_3$): δ 7.48 – 7.35 (m, 5H), 3.65 (t, *J* = 7.0 Hz, 2H), 3.43 (t, *J* = 6.6 Hz, 2H), 2.01 – 1.84 (m, 9H).

N-(4-chlorobenzoyl)pyrrolidine (7d)
Column chromatography (CH_2Cl_2:EtOAc 25:10) giving colorless oil. Yield 105 mg 71%.
^{1}H NMR: δ 7.47 (d, *J* = 8.5 Hz, 2H), 7.38 (d, *J* = 8.5 Hz, 2H), 3.63 (t, *J* = 6.9 Hz, 2H), 3.41 (t, *J* = 6.6Hz, 2H), 1.99 – 1.93 (m, 2H), 1.91 – 1.86 (m, 2H).

(4-bromophenyl)(pyrrolidin-1-yl)methanone (7e)
Column chromatography (CH_2Cl_2:EtOAc 35:10) giving a white crystals. Yield 72 mg, 57 %. ^{1}H NMR: δ 7.53 (d, *J* = 8.5 Hz, 2H), 7.40 (d, *J* = 8.5 Hz, 2H), 3.41 (t, J = 6.6 Hz, 2H), 3.62 (t, J = 6.9 Hz, 2H), 2-1.85 (m, 4H).

***N*,*N*-diethyl-4-nitrobenzamide (8)**
Column chromatography (Hexane:EtOAc 80:20) giving a yellow solid. Yield 49 mg, 34%.
^{1}H NMR (400 MHz, $CDCl_3$): δ 8.28 (d, *J* = 8.6 Hz, 2H), 7.54 (d, *J* = 8.6 Hz, 2H), 3.58 (q, *J* = 7.2 Hz, 4H), 3.22 (q, *J* = 7.2 Hz, 4H), 1.13 (td, *J* = 7.0, 2.5 Hz, 6H).

***N*,*N*-dibutyl-4-nitrobenzamide (8a)**
Column chromatography (Hexane:EtOAc 80:20) giving a yellow oil. Yield 83 mg, 43%.
^{1}H NMR: δ 8.28 (d, *J* = 8.8 Hz, 2H), 7.93 (d, *J* = 8.8 Hz, 2H), 3.50 – 3.47 (m, 2H), 3.17 (dt, *J* = 24.6, 7.4 Hz, 2H), 1.66 – 1.59 (m, 4H), 1.46 – 1.39 (m, 4H), 0.97 (t, *J* = 7.3 Hz, 6H).

N-(4-Nitrobenzoyl)morpholine (8b)
Column chromatography (CH2Cl2:EtOAc 80:10) giving a yellow crystals. ^{1}H NMR: δ 8.30 (d, *J* = 8.6 Hz, 2H), 7.59 (d, *J* = 8.6 Hz, 2H), 3.81 (s, 4H), 3.64 (s, 2H), 3.40 (s, 2H).

Piperazine-1,4-diylbis((4-nitrophenyl)methanone) (8c)
Column chromatography (CH2Cl2:EtOAc 80:10) giving a yellow crystals Yield 40.6 mg, 32%. ^{1}H NMR: δ 8.28 (d, *J* = 8.6 Hz, 2H), 7.58 (d, *J* = 8.7 Hz, 2H), 3.86 – 3.72 (m, 2H), 3.35 (t, *J* = 4.9 Hz, 2H), 2.97 (d, *J* = 5.5 Hz, 2H), 2.83 (t, *J* = 4.9 Hz, 2H).

NMR Spectra

2,5-dimethyl-3-(1*H*-pyrrol-2-yl)cyclohexa-2,5-diene-1,4-dione (4)

^{1}H NMR (400 MHz, $CDCl_3$)

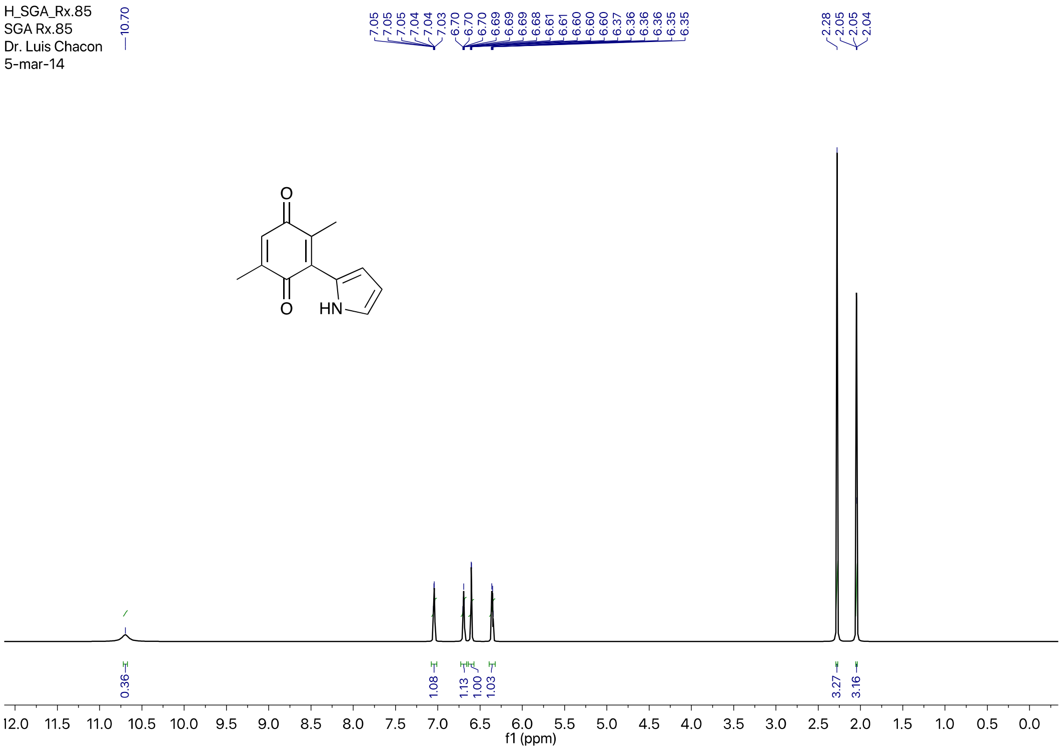

^{13}C NMR (101 MHz, $CDCl_3$)

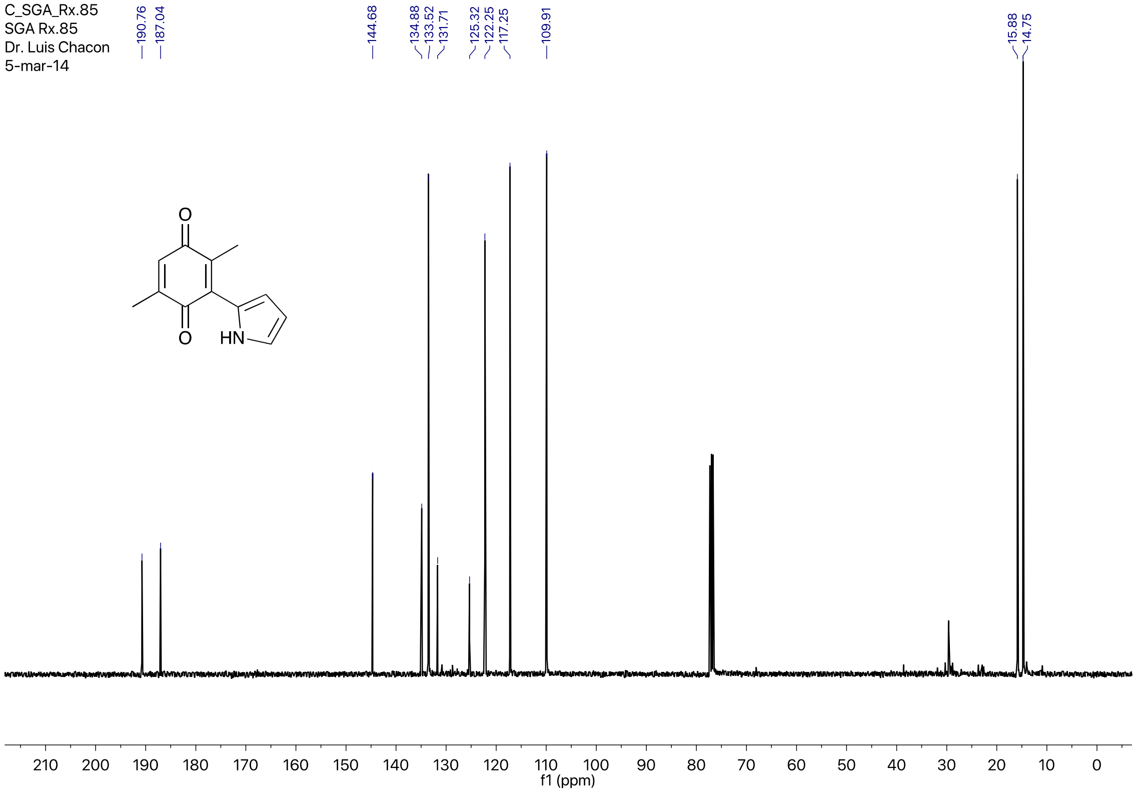

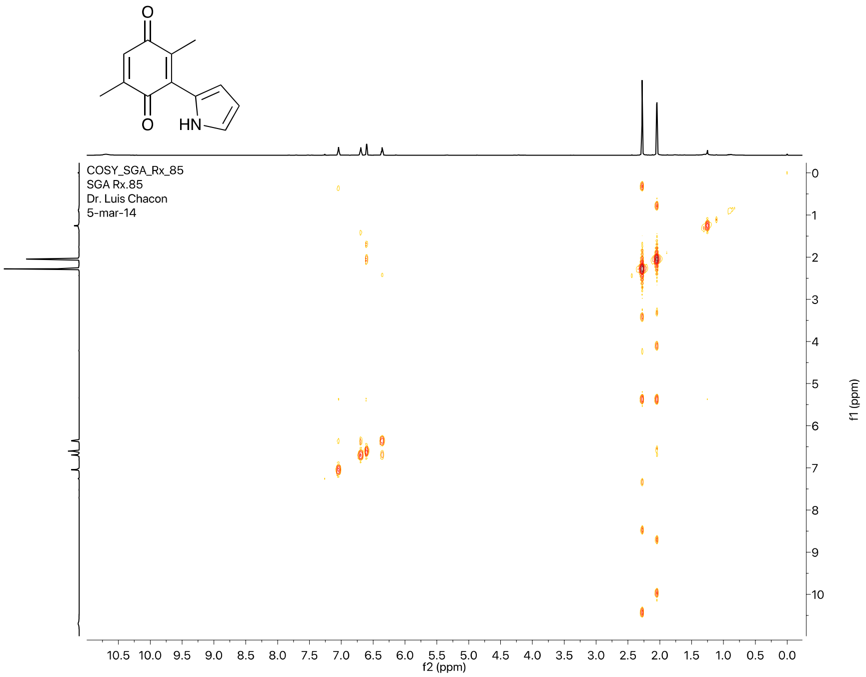

Cosy NMR (400 MHz, $CDCl_3$)

Hetcor NMR (400 MHz, $CDCl_3$)

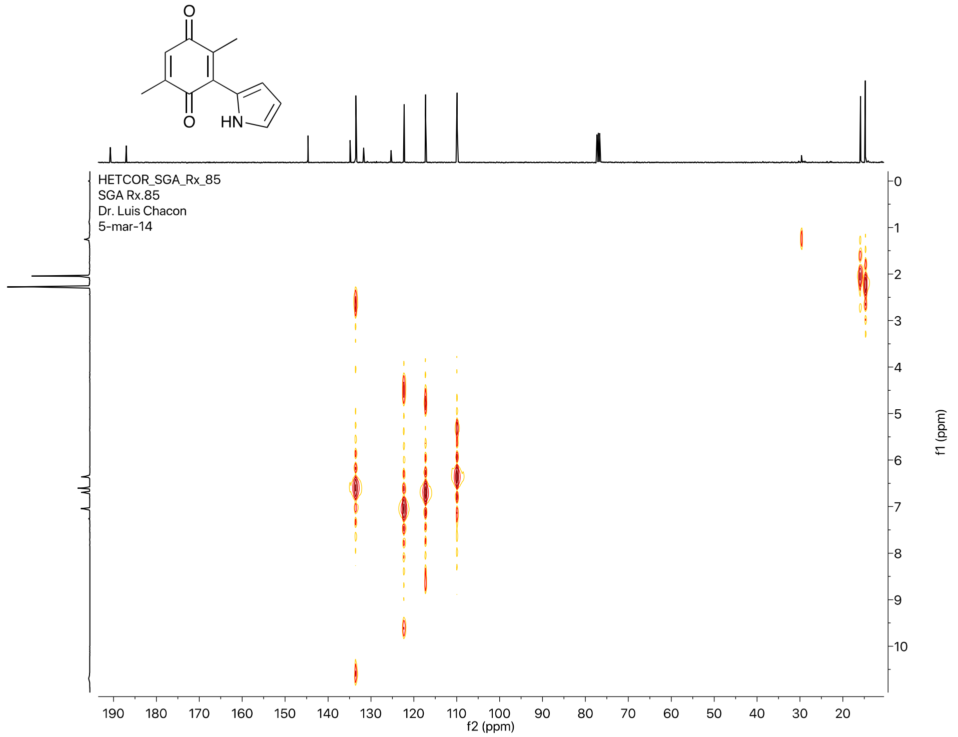

Noesy NMR (400 MHz, $CDCl_3$)

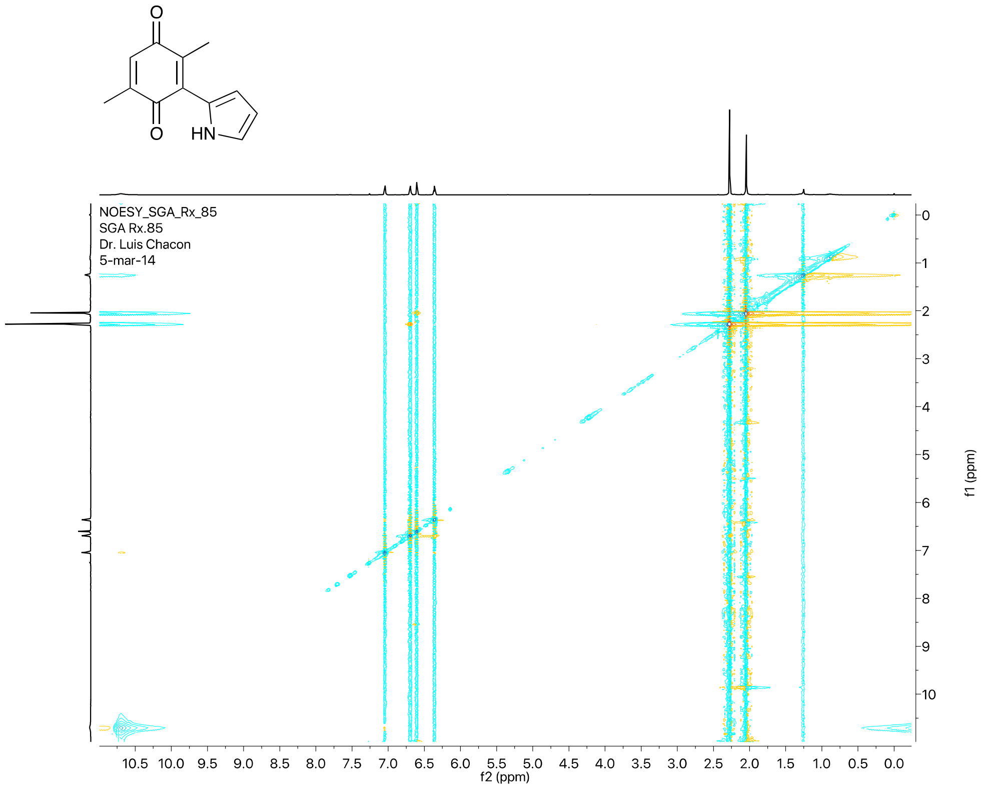

Dept NMR (400 MHz, $CDCl_3$)

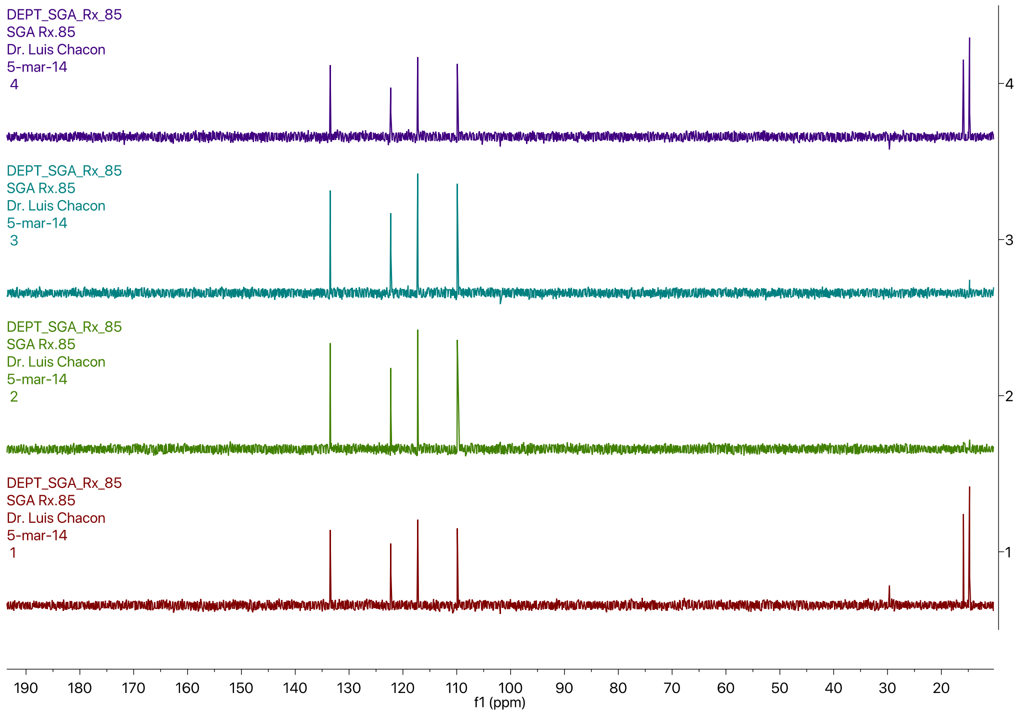

Mass spectrum

File: QDP_PIRROL_LCG **Date Run: 08-19-2014 (Time Run: 12:00:41)**
Sample: QUINONA_PIRROL
Instrument: JEOL GCmate
Inlet: Direct Probe **Ionization mode: EI+**

Scan: 46 **R.T.: .61**
Base: m/z 181; 41.7%FS TIC: 1382848 **#Ions: 100**

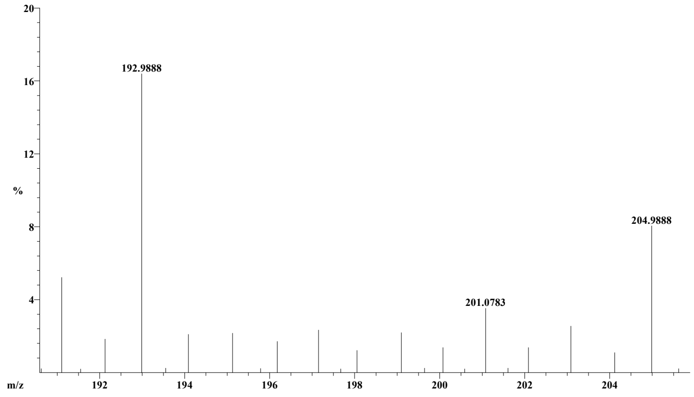

Selected Isotopes : C H $N_{0-1}O_{0-2}$ **Error Limit : 5 ppm**

Measured Mass	% Base	Formula	Calculated Mass	Error
201.0783	3.5%	$C_{12}H_{11}N O_2$	201.0790	-3.4

3-(5-chloro-1*H*-pyrrol-2-yl)-2,5-dimethylcyclohexa-2,5-diene-1,4-dione (4a)

^{1}H NMR (400 MHz, CDC_{l_3})

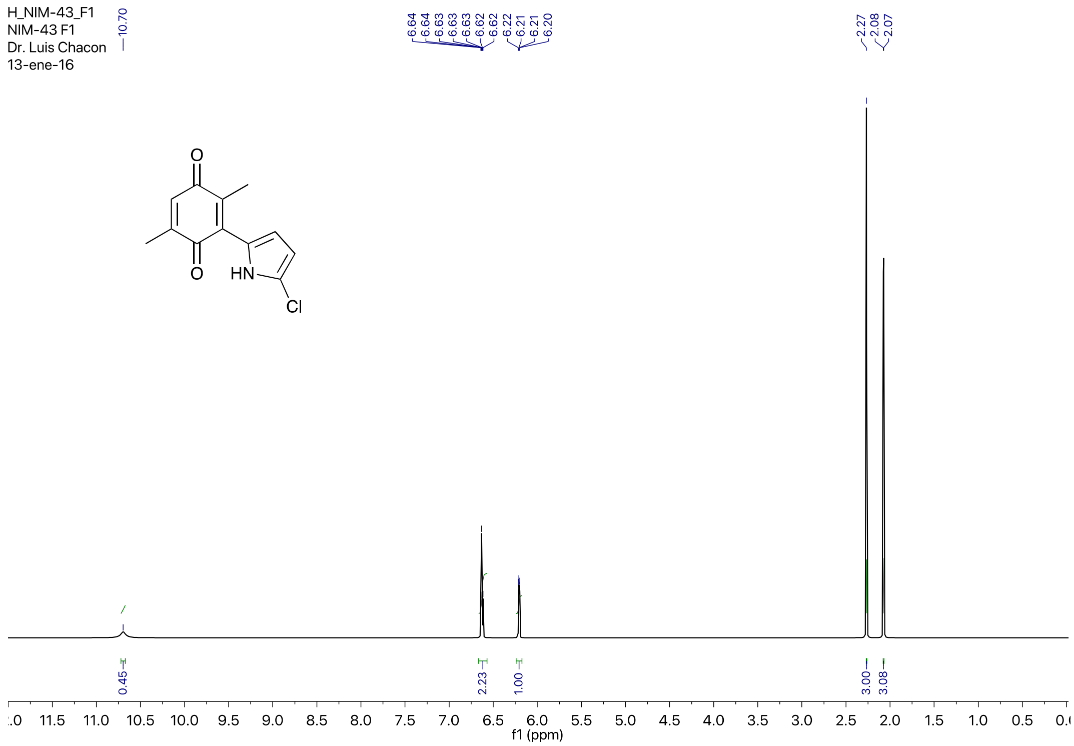

^{13}C NMR (101 MHz, $CDCl_3$)

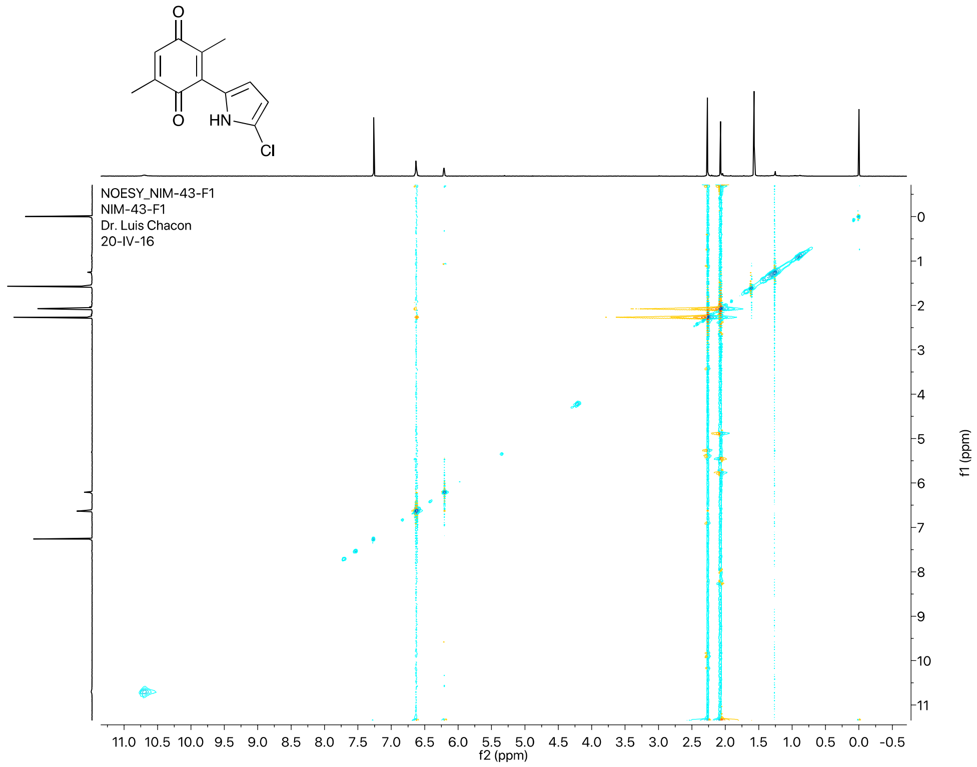

Cosy NMR (400 MHz, $CDCl_3$

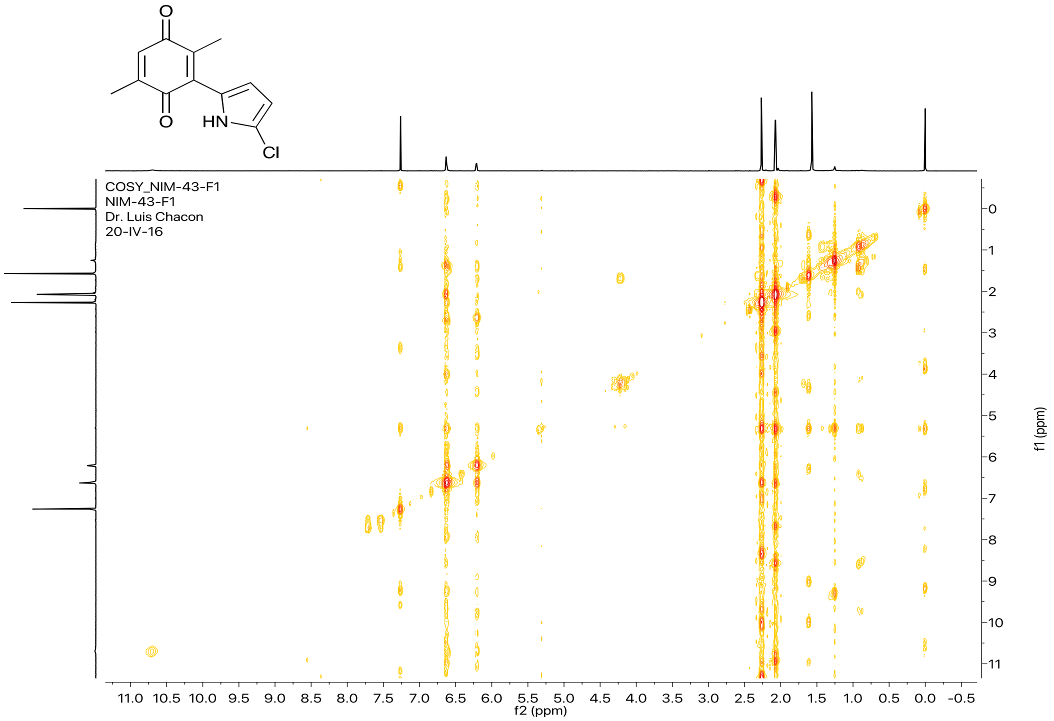

Hetcor NMR (400 MHz, $CDCl_3$)

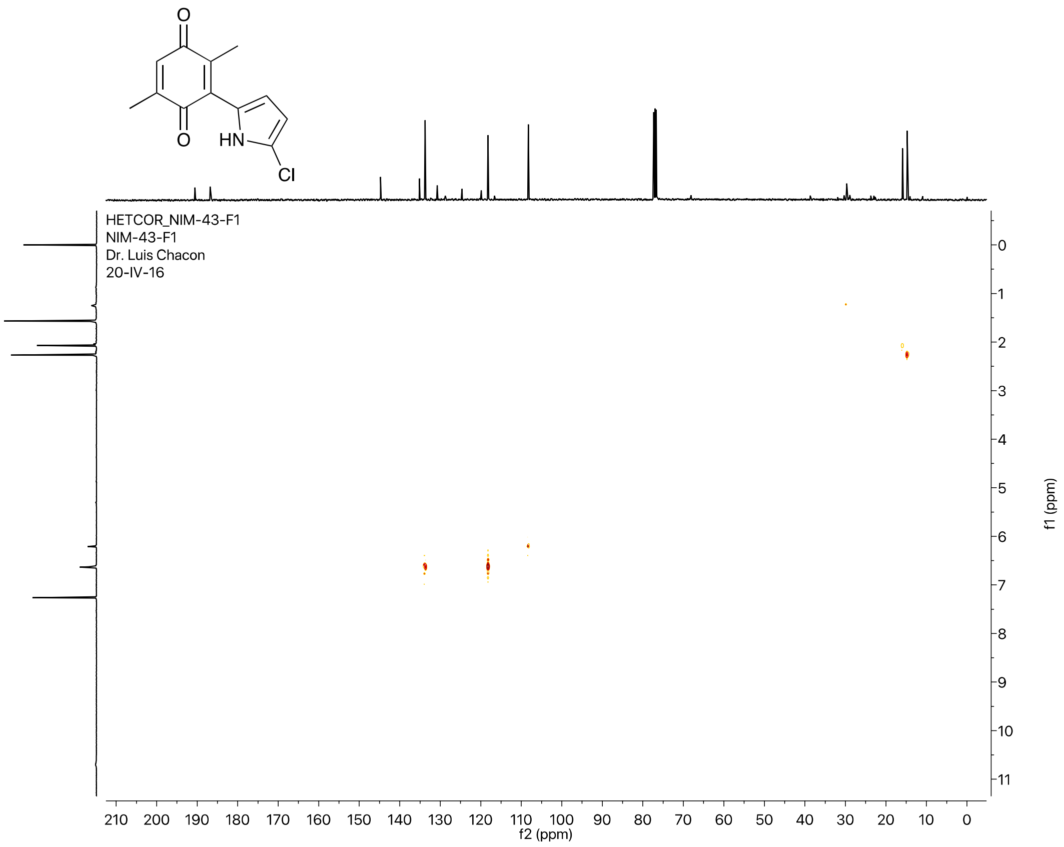

Noesy NMR (400 MHz, $CDCl_3$)

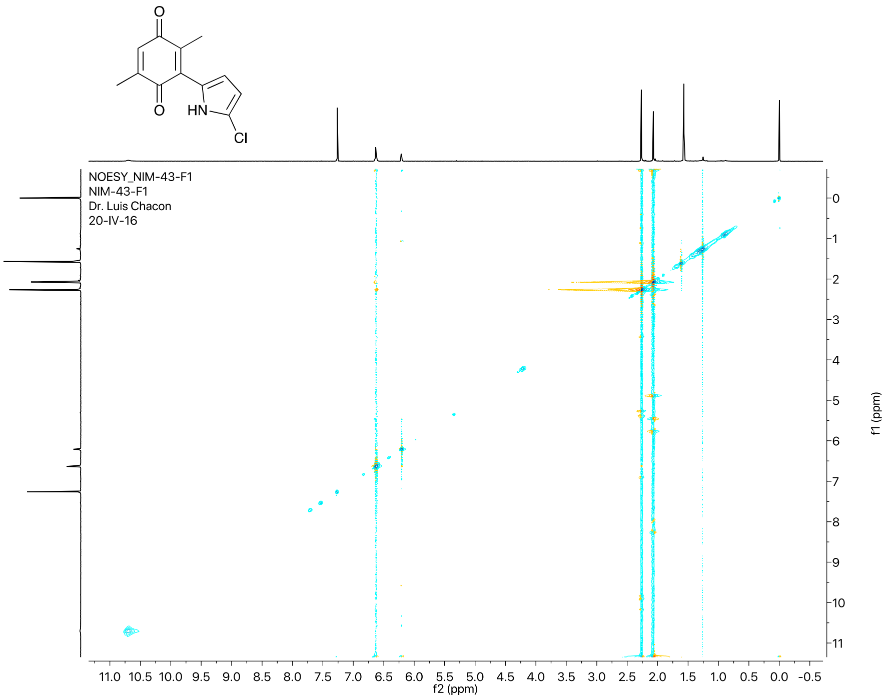

Dept NMR (400 MHz, $CDCl_3$)

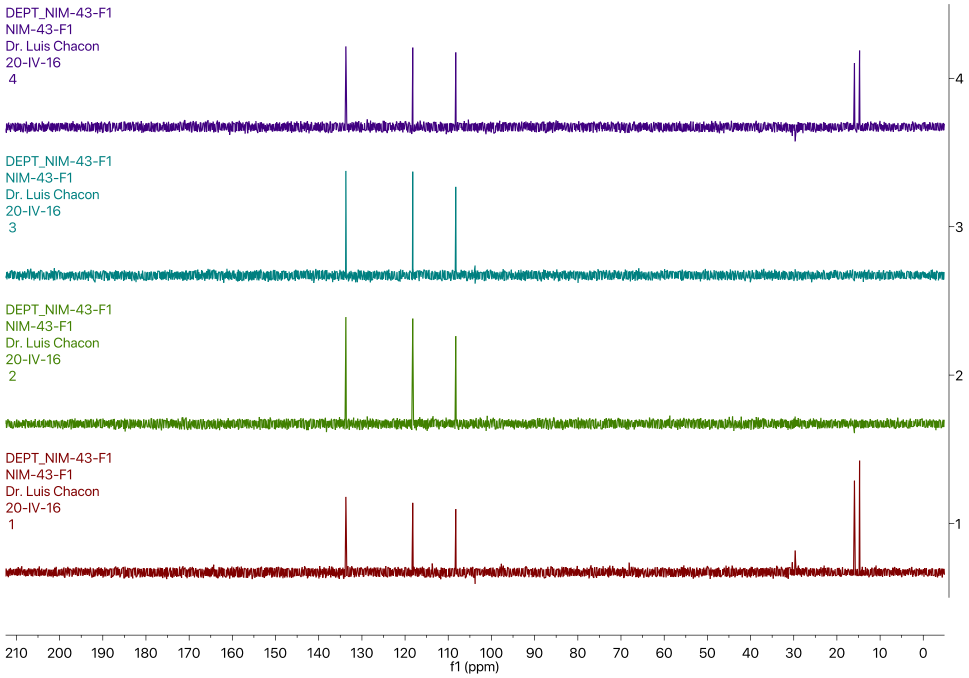

Mass spectrum

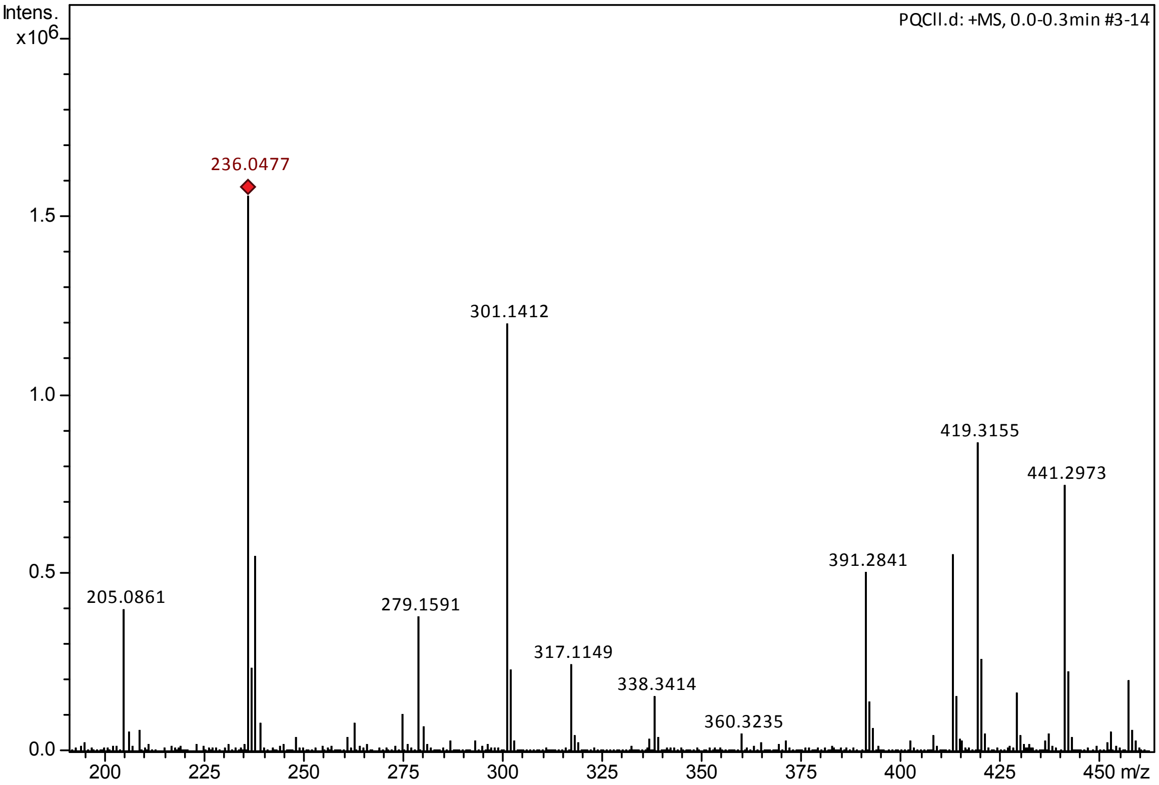

2,5-dimethyl-3-(5-nitro-1*H*-pyrrol-2-yl)cyclohexa-2,5-diene-1,4-dione (4b)

^{1}H NMR (400 MHz, $CDCl_3$)

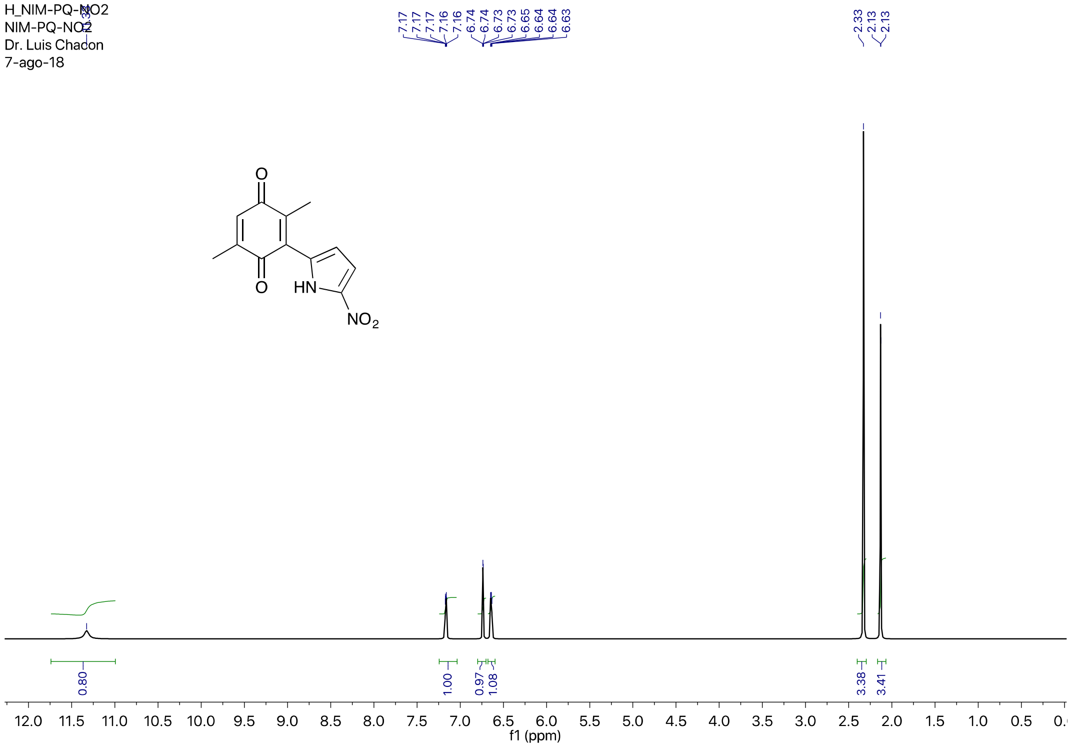

^{13}C NMR (101 MHz, $CDCl_3$)

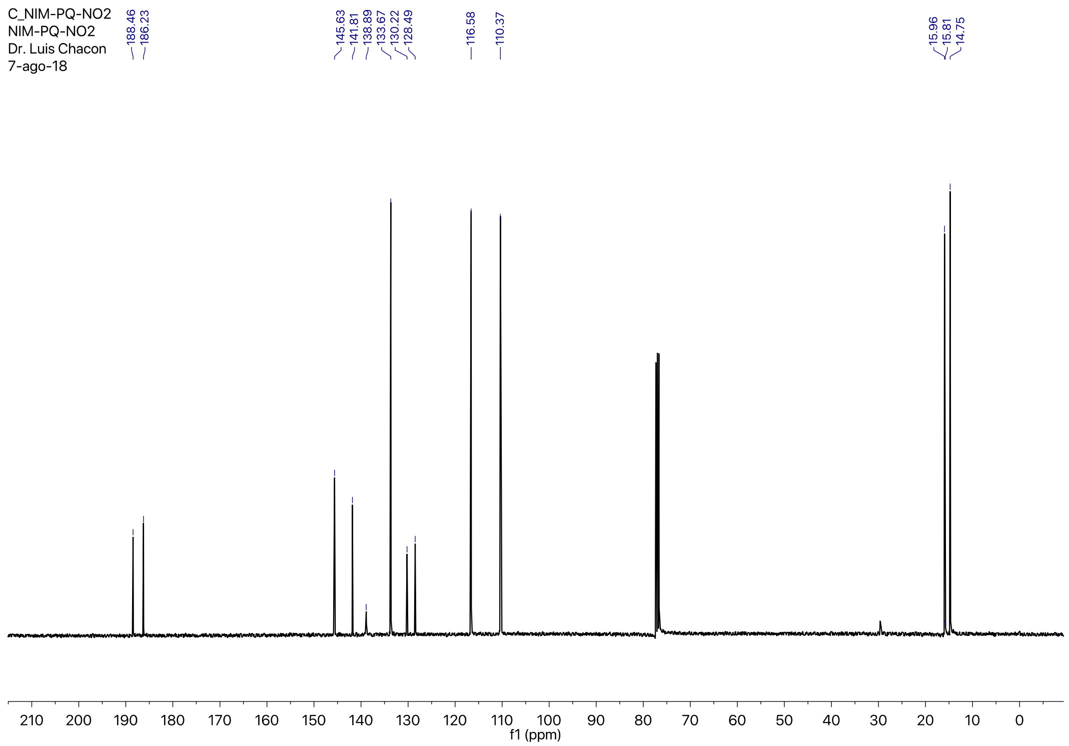

Cosy NMR (400 MHz, $CDCl_3$)

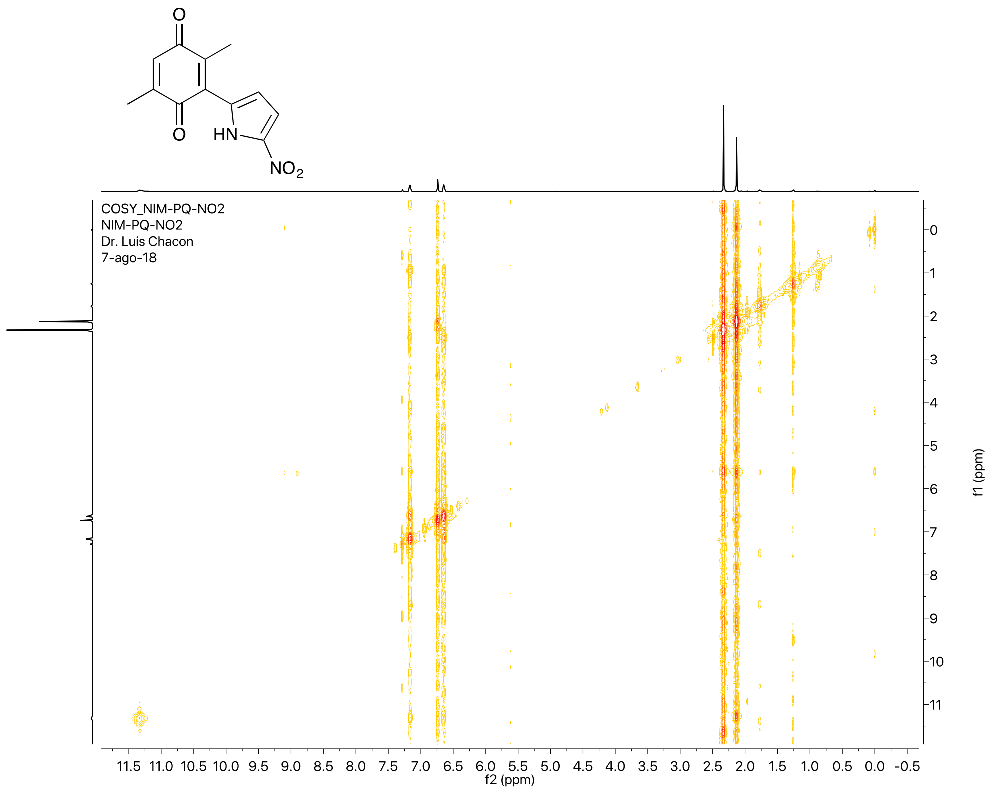

Hetcor NMR (400 MHz, $CDCl_3$)

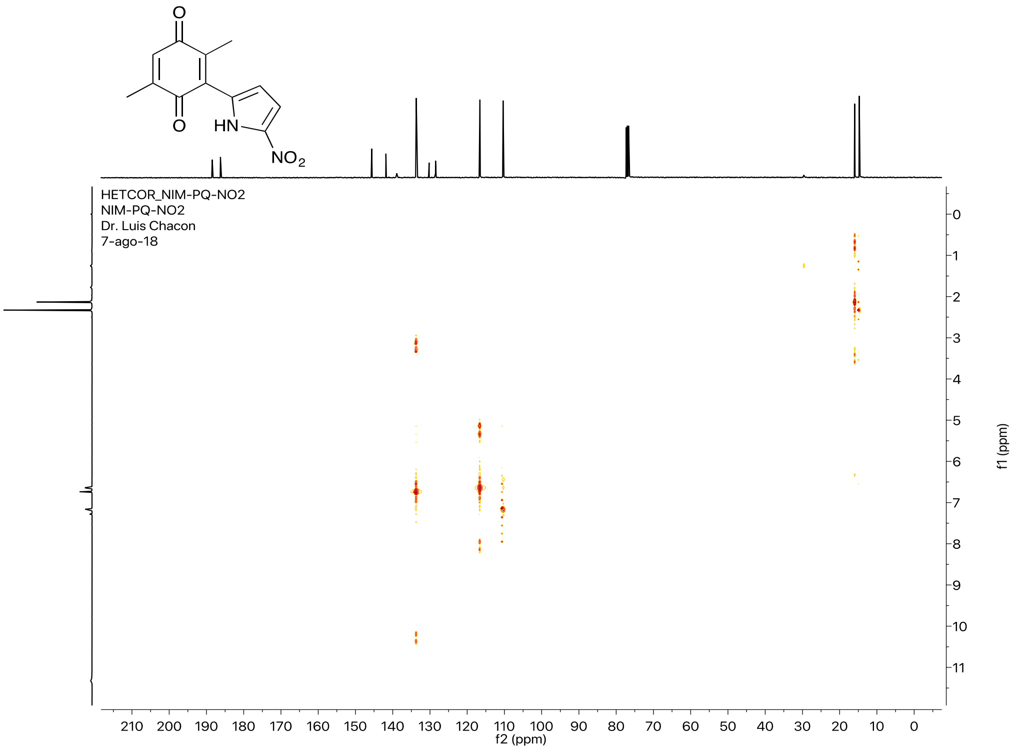

Noesy NMR (400 MHz, $CDCl_3$)

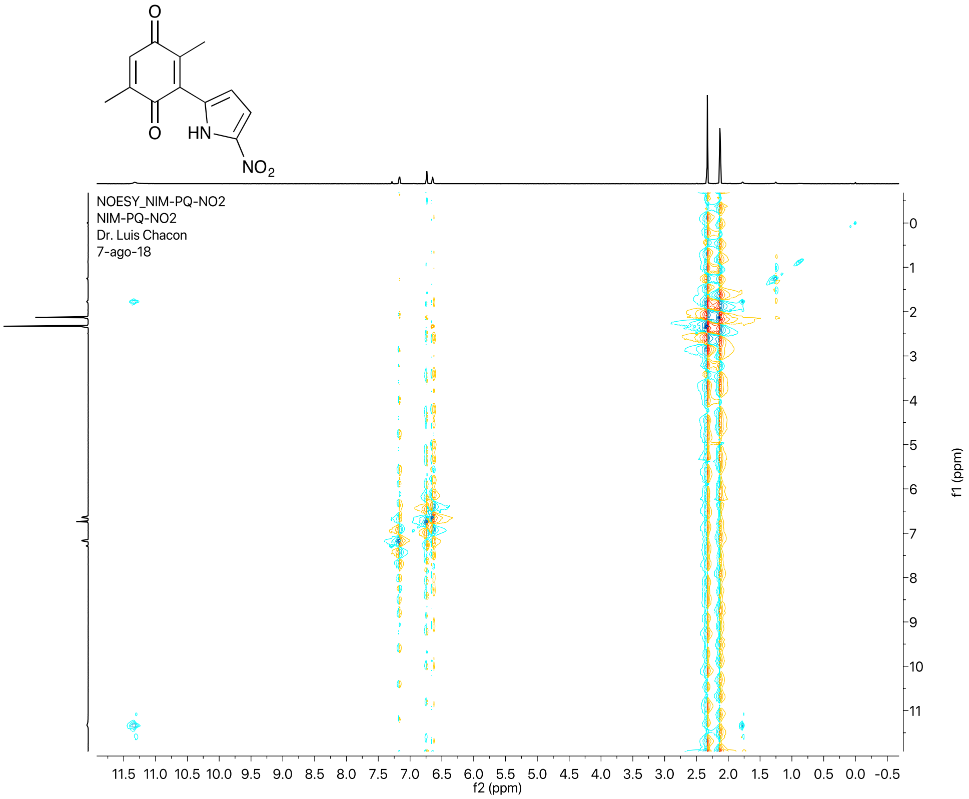

Dept NMR (400 MHz, $CDCl_3$)

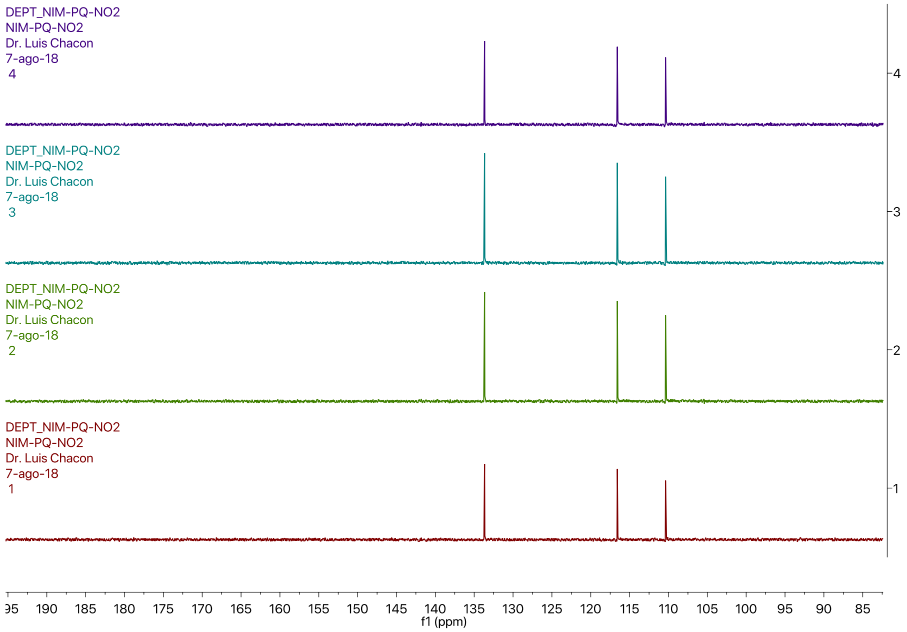

Mass spectrum

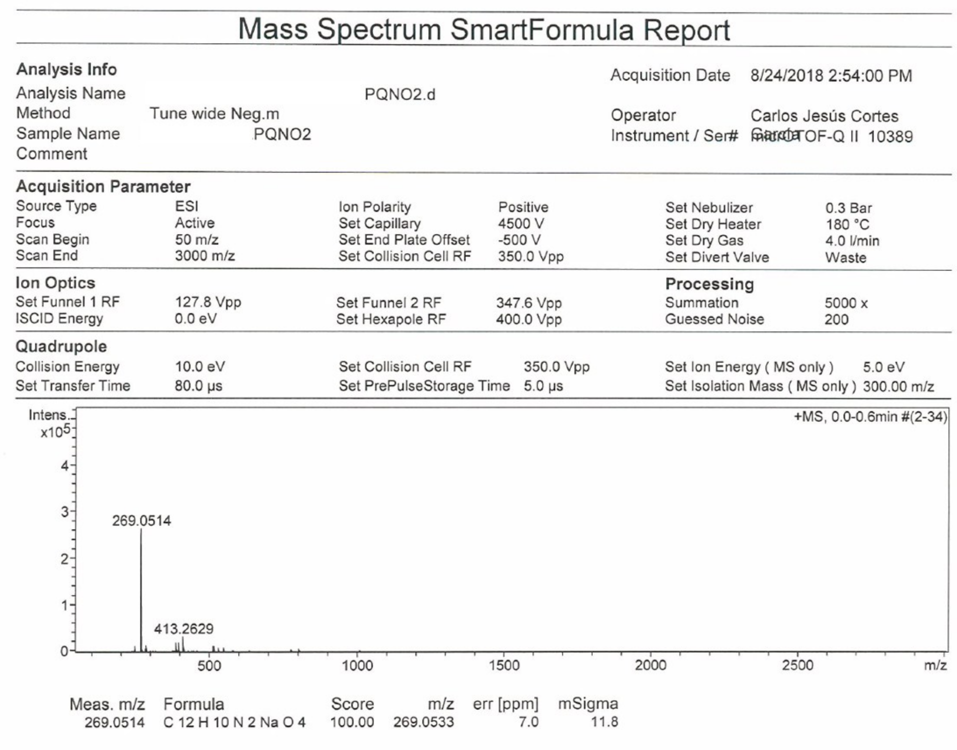

Mass Spectrum SmartFormula Report

Analysis Info

Analysis Name	PQNO2.d	Acquisition Date	8/24/2018 2:54:00 PM
Method	Tune wide Neg.m	Operator	Carlos Jesús Cortes Garcia
Sample Name	PQNO2	Instrument / Ser#	micrOTOF-Q II 10389
Comment			

Acquisition Parameter

Source Type	ESI	Ion Polarity	Positive	Set Nebulizer	0.3 Bar
Focus	Active	Set Capillary	4500 V	Set Dry Heater	180 °C
Scan Begin	50 m/z	Set End Plate Offset	-500 V	Set Dry Gas	4.0 l/min
Scan End	3000 m/z	Set Collision Cell RF	350.0 Vpp	Set Divert Valve	Waste

Ion Optics / **Processing**

Set Funnel 1 RF	127.8 Vpp	Set Funnel 2 RF	347.6 Vpp	Summation	5000 x
ISCID Energy	0.0 eV	Set Hexapole RF	400.0 Vpp	Guessed Noise	200

Quadrupole

Collision Energy	10.0 eV	Set Collision Cell RF	350.0 Vpp	Set Ion Energy (MS only)	5.0 eV
Set Transfer Time	80.0 µs	Set PrePulseStorage Time	5.0 µs	Set Isolation Mass (MS only)	300.00 m/z

Meas. m/z	Formula	Score	m/z	err [ppm]	mSigma
269.0514	C 12 H 10 N 2 Na O 4	100.00	269.0533	7.0	11.8

2,5-dimethyl-3-(5-thiocyanato-1*H*-pyrrol-2-yl)cyclohexa-2,5-diene-1,4-dione (4c).
^{1}H NMR (400 MHz, $CDCl_3$)

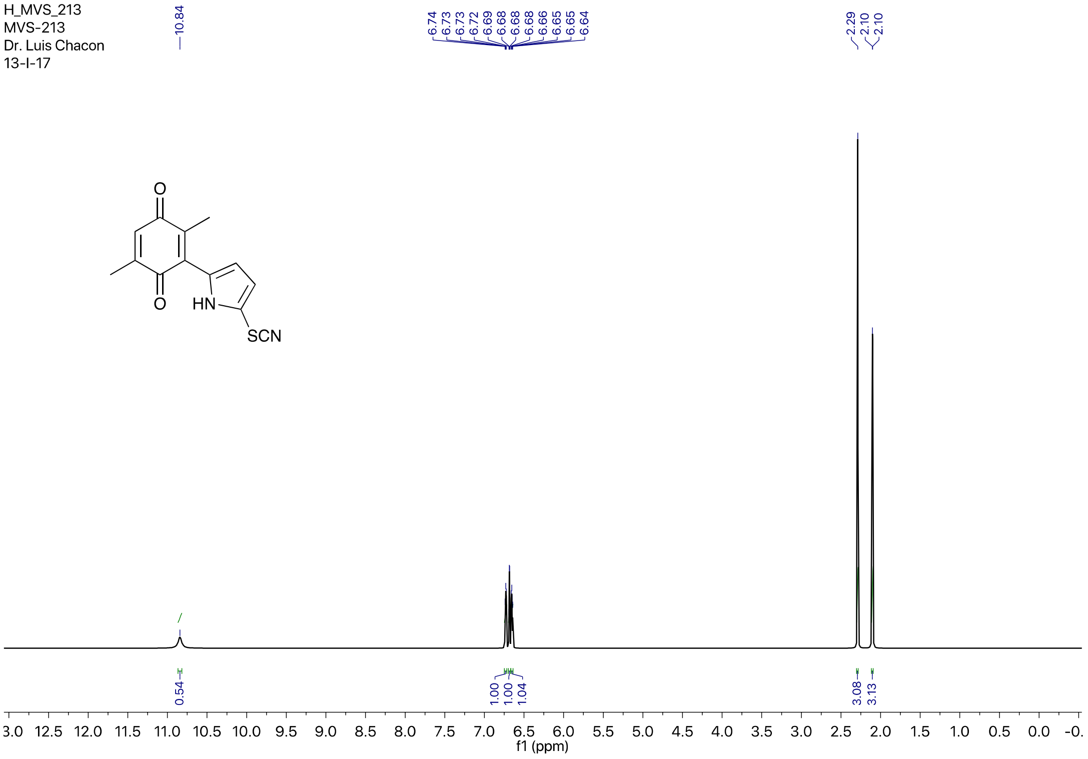

^{13}C NMR (101 MHz, $CDCl_3$)

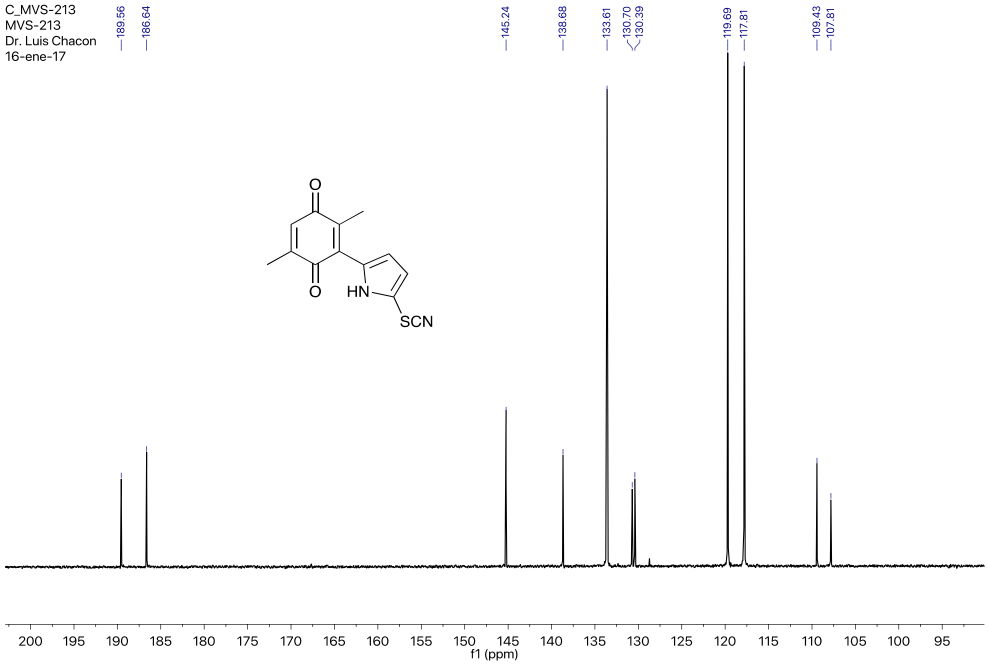

Cosy NMR (400 MHz, $CDCl_3$)

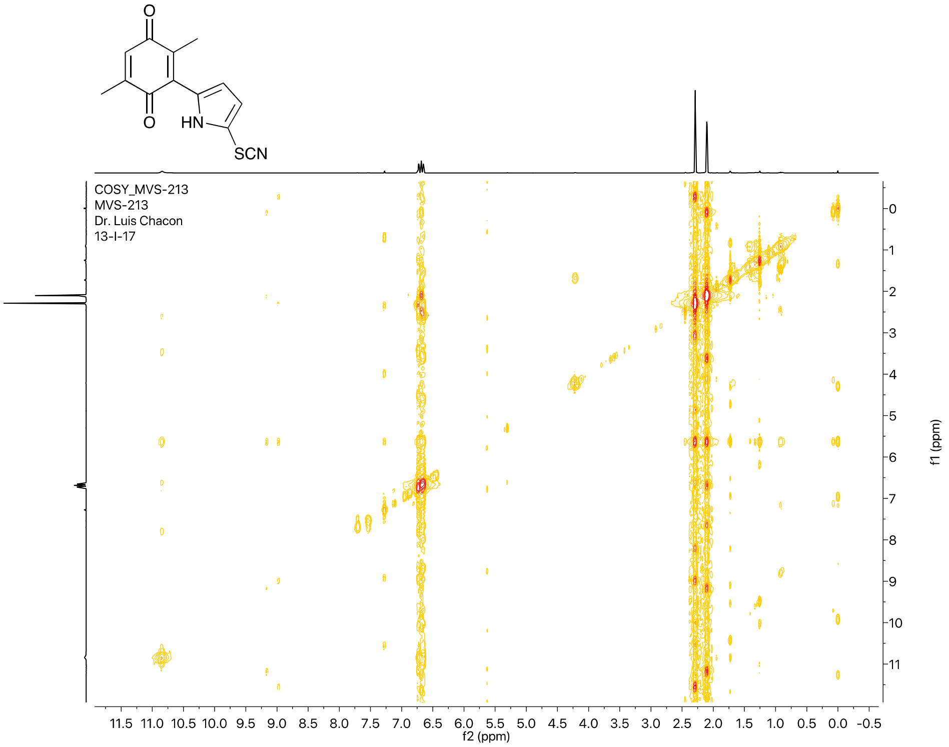

Hetcor NMR (400 MHz, $CDCl_3$)

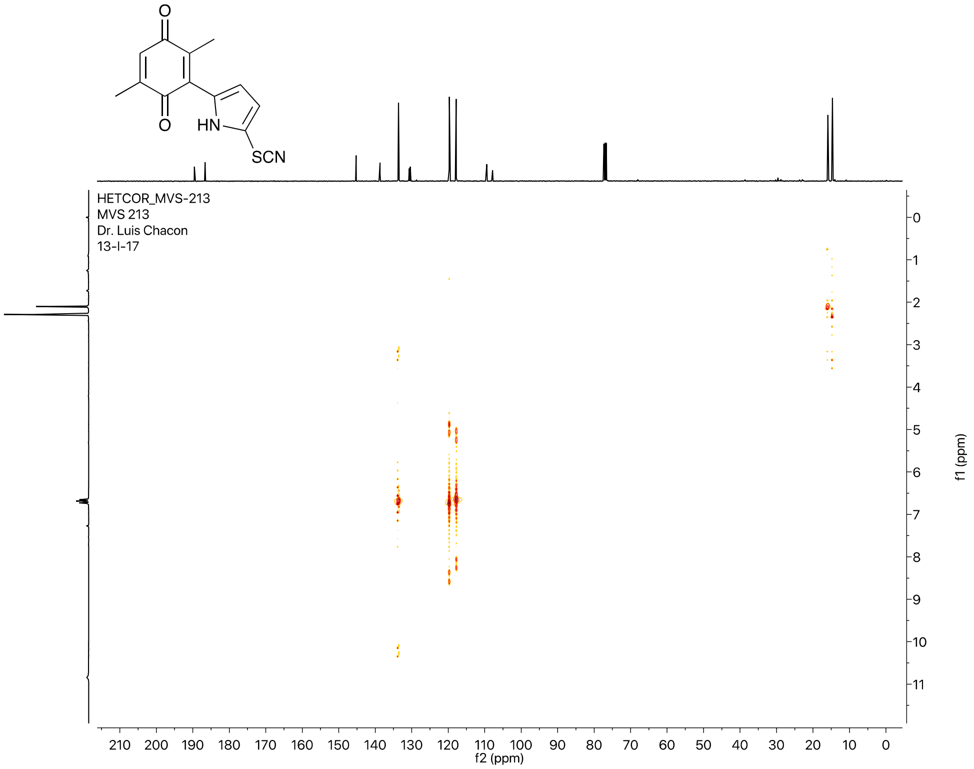

Noesy NMR (400 MHz, $CDCl_3$)

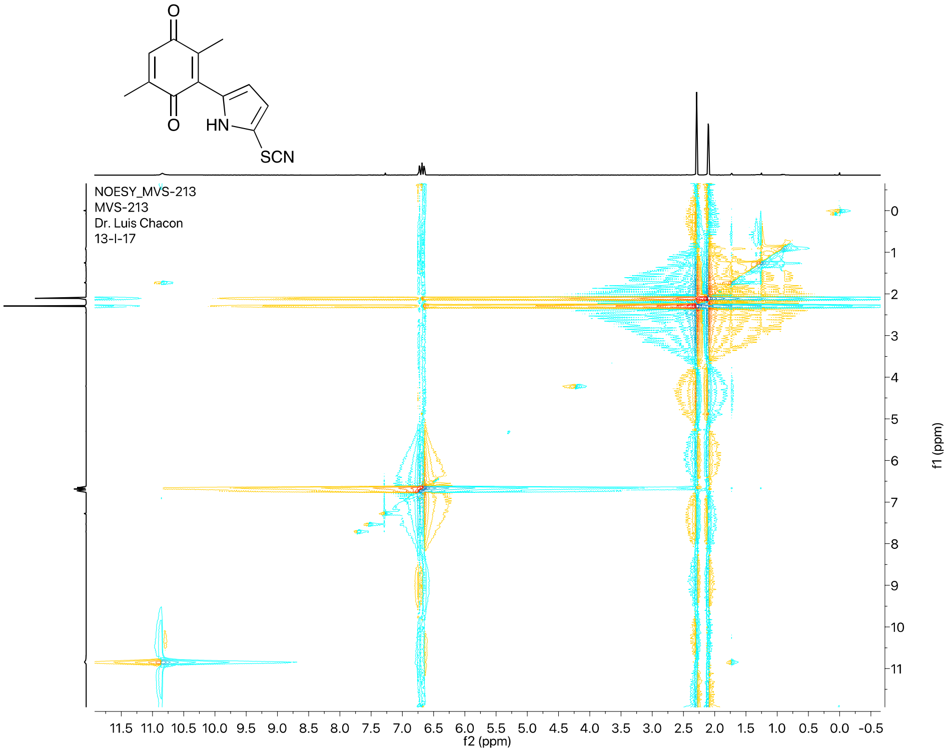

Dept NMR (400 MHz, $CDCl_3$)

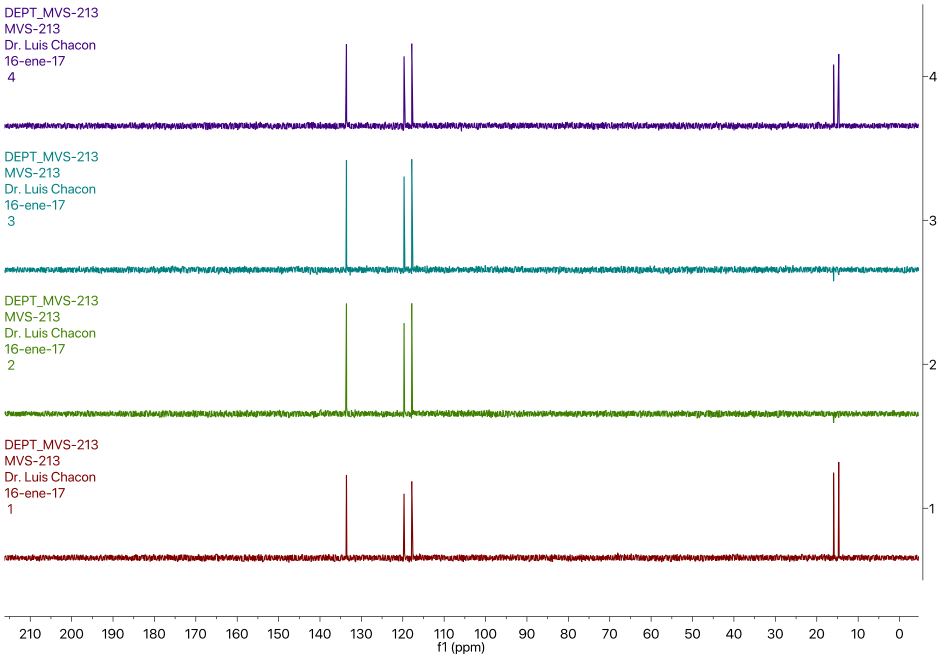

Mass spectrum

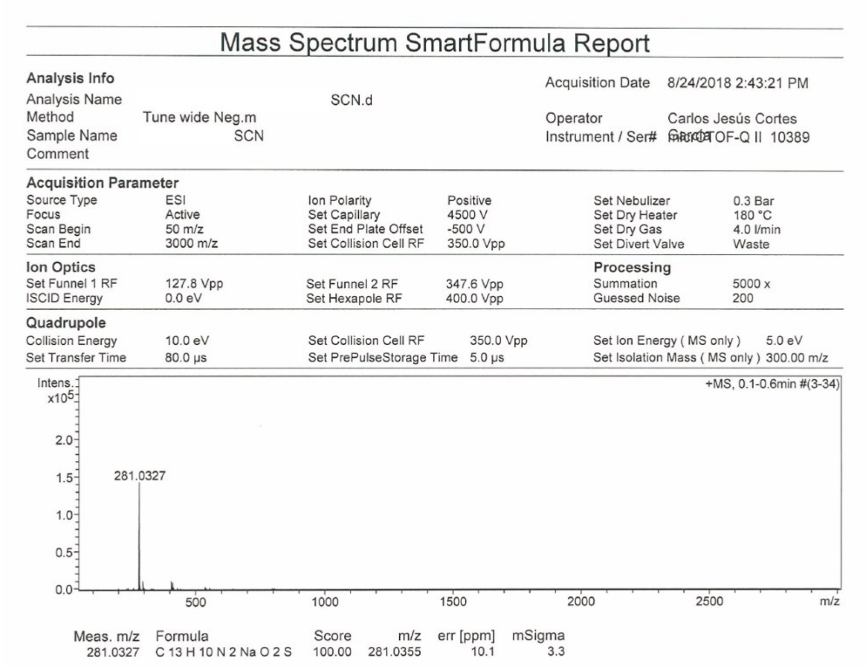

2-hydroxy-6-methyl-3-(6-methylheptan-2-yl)-5-(1*H*-pyrrol-2-yl)cyclohexa-2,5-diene-1,4-dione (5)

^{1}H NMR (400 MHz, $CDCl_3$)

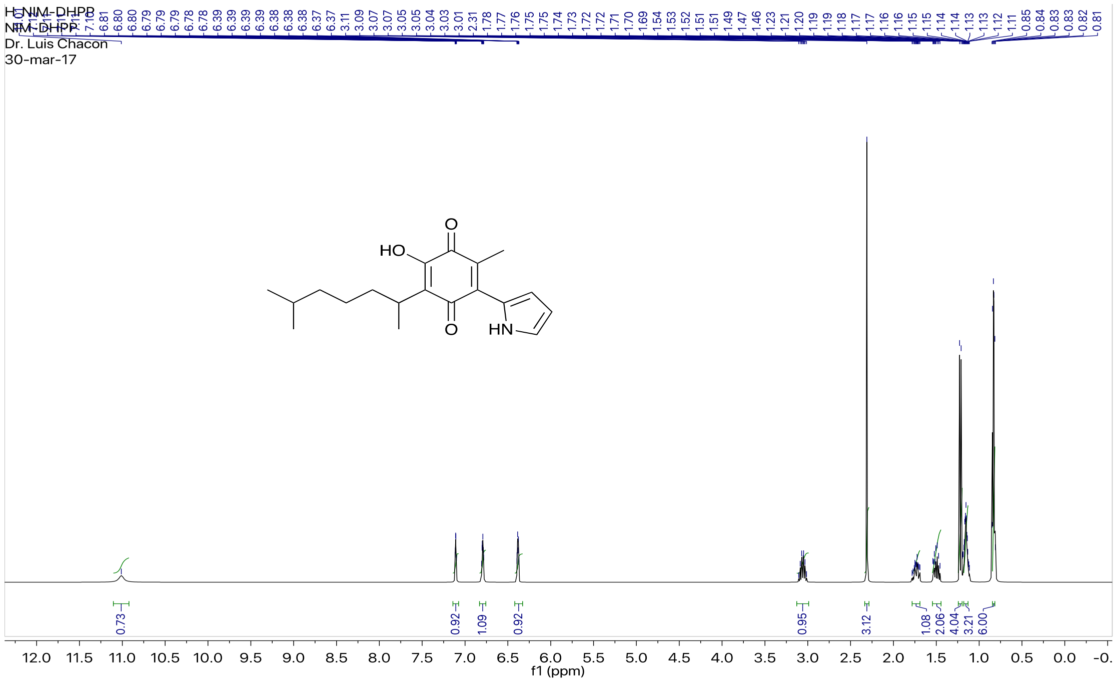

^{13}C NMR (101 MHz, $CDCl_3$)

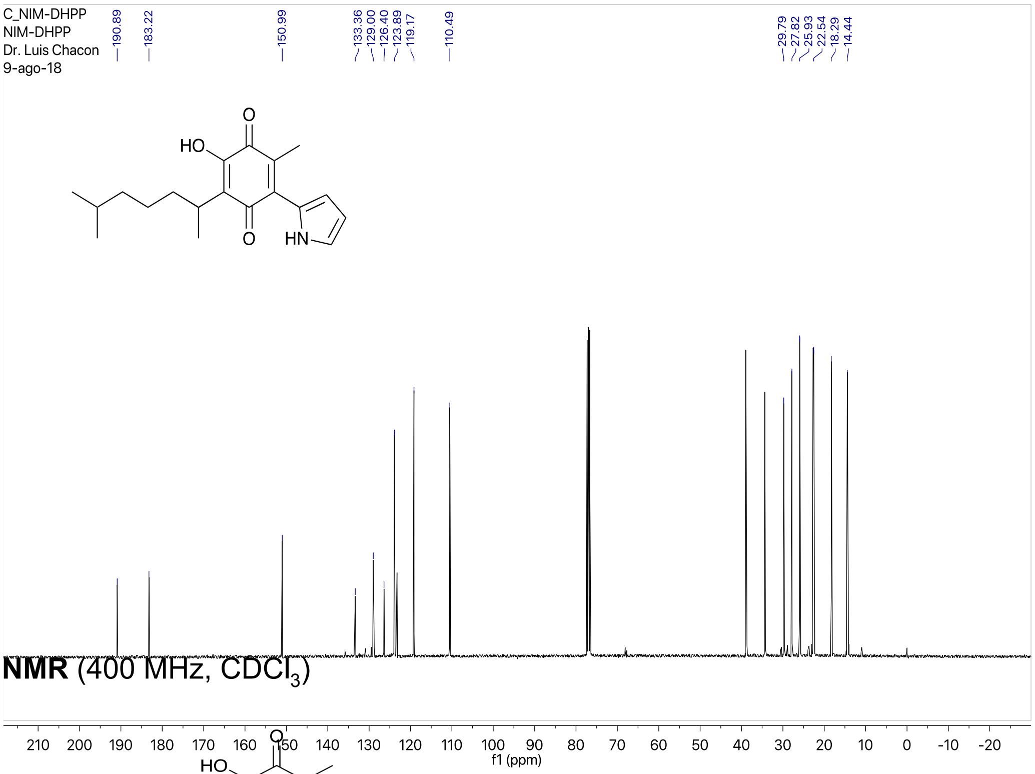

Cosy NMR (400 MHz, $CDCl_3$)

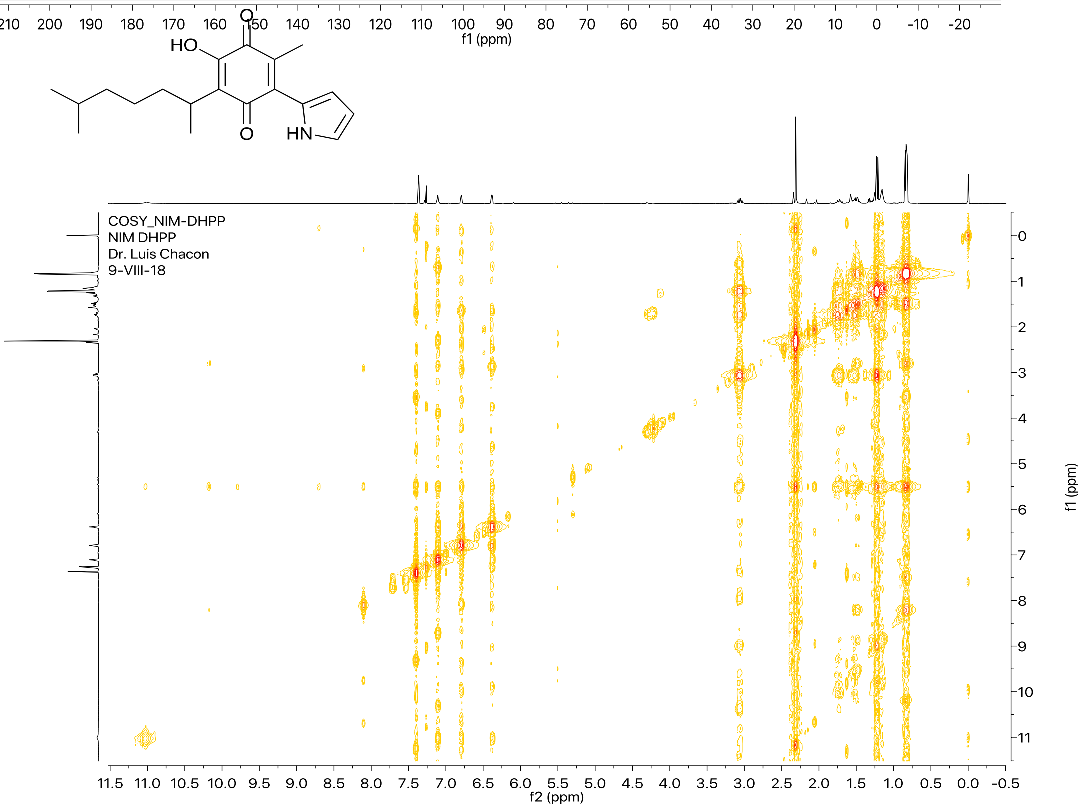

Hetcor NMR (400 MHz, $CDCl_3$)

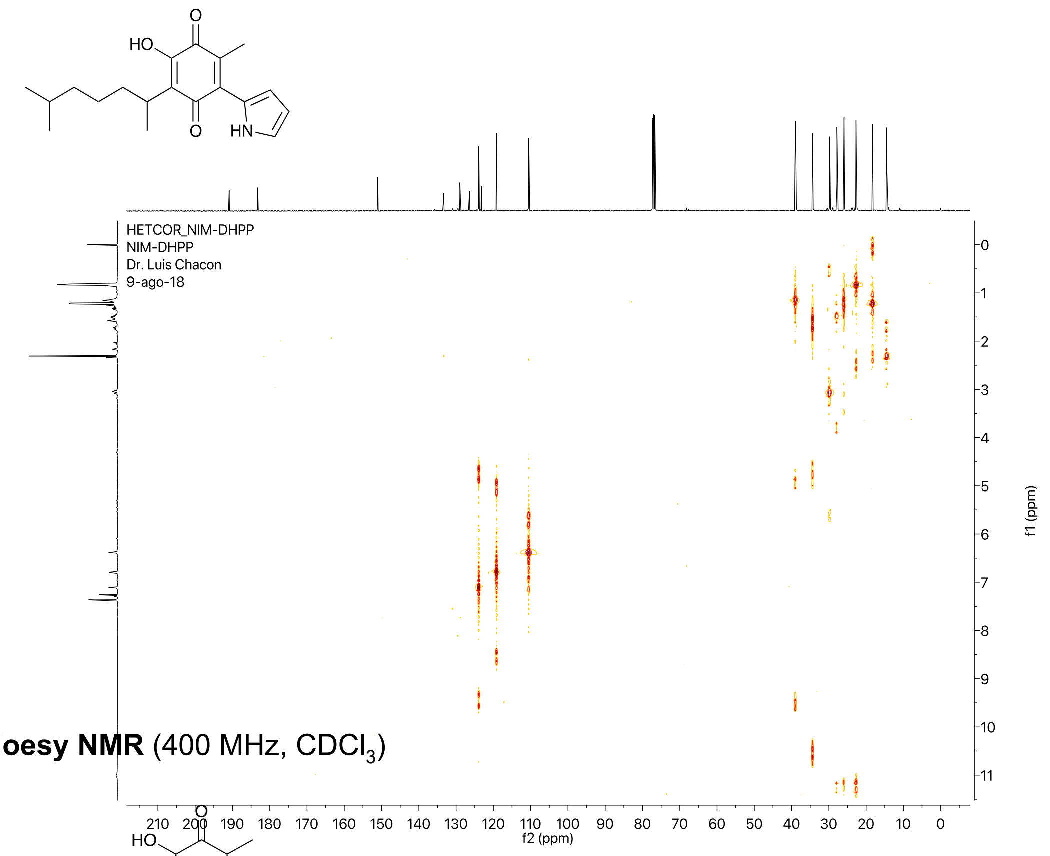

Noesy NMR (400 MHz, $CDCl_3$)

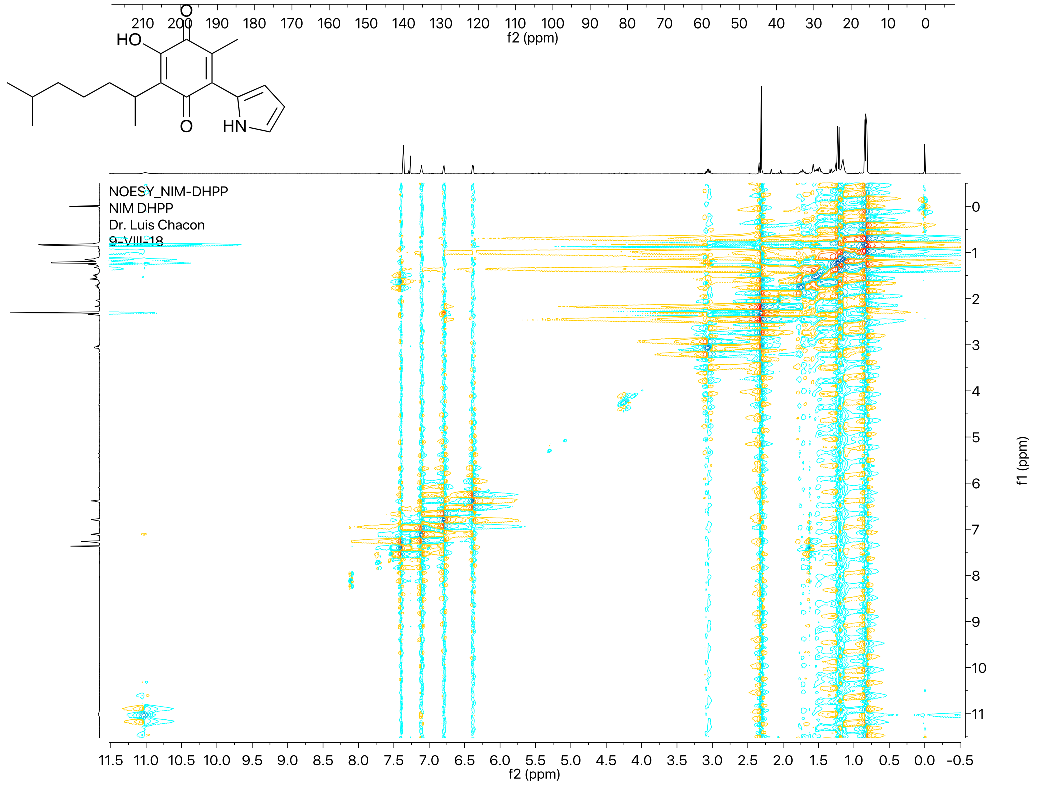

Dept NMR (400 MHz, $CDCl_3$)

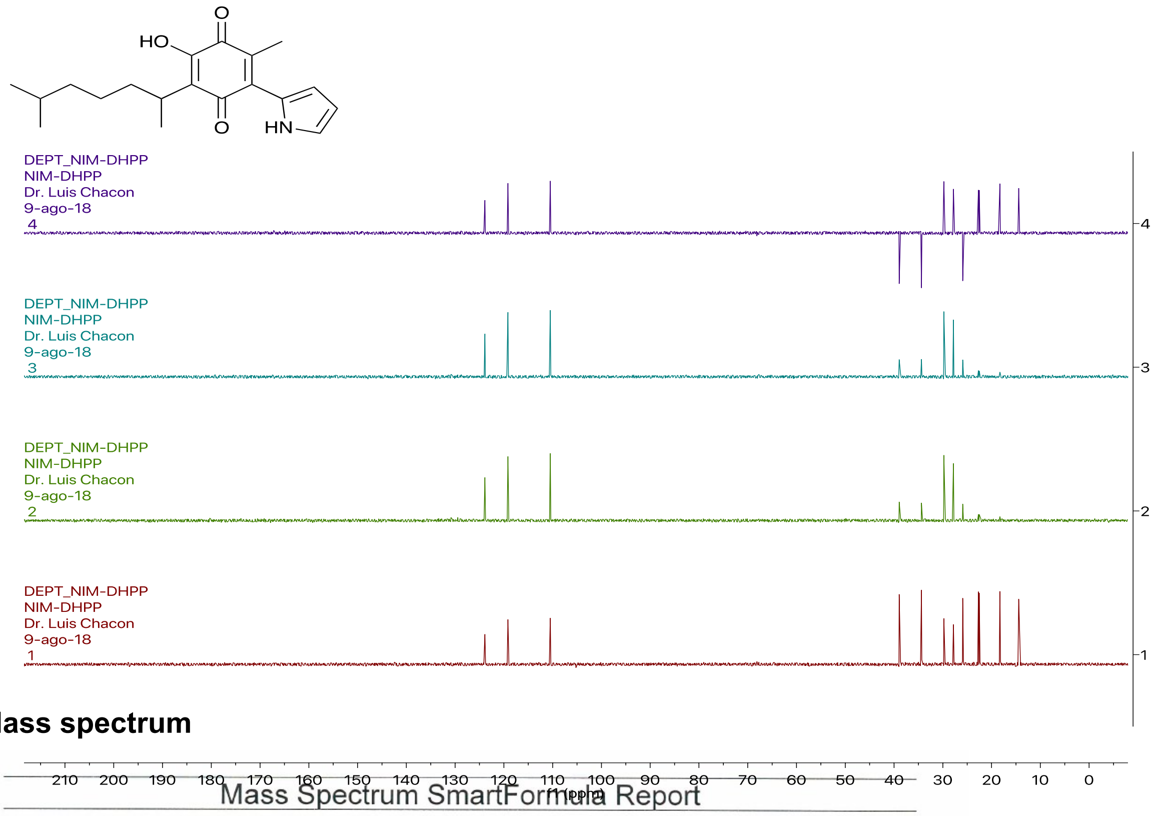

Mass spectrum

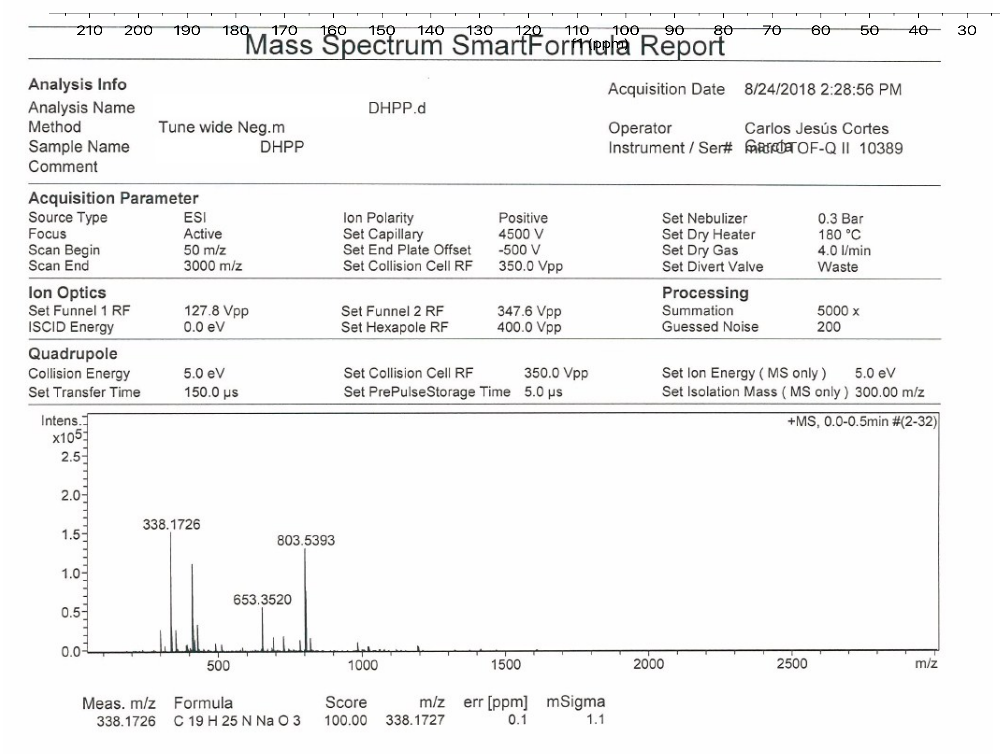

(4-nitrophenyl)(pyrrolidin-1-yl)methanone (7a)

^{1}H NMR (400 MHz, $CDCl_3$)

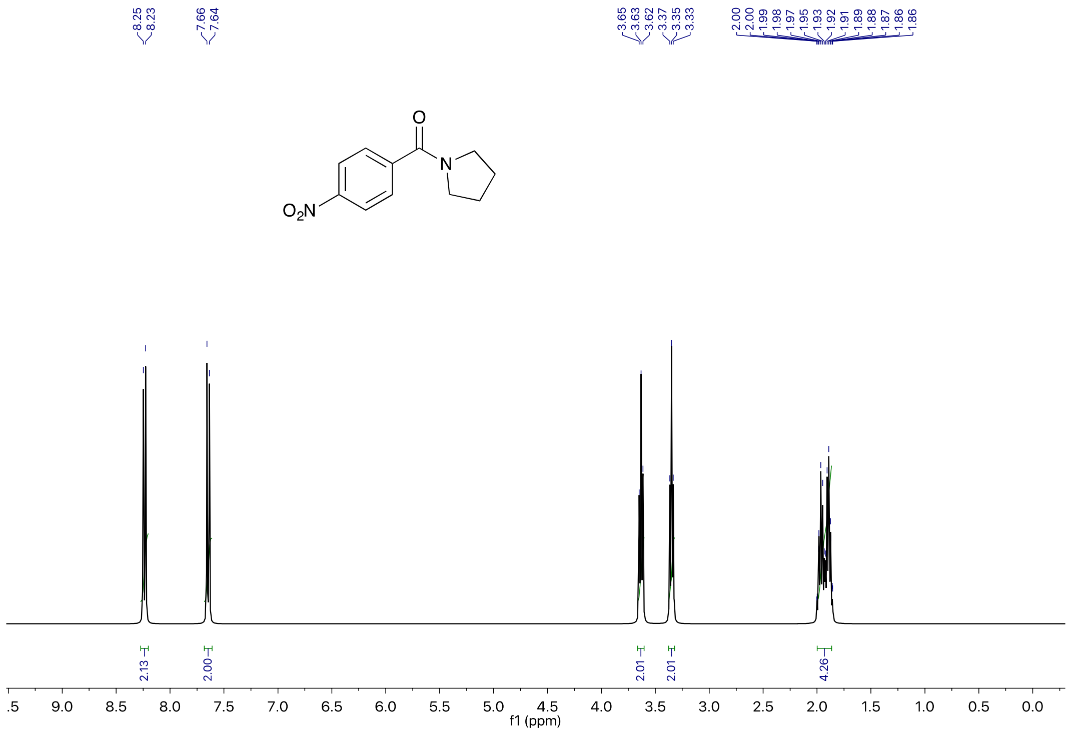

(4-methoxyphenyl)(pyrrolidin-1-yl)methanone (7b)
^{1}H NMR (400 MHz, $CDCl_3$)

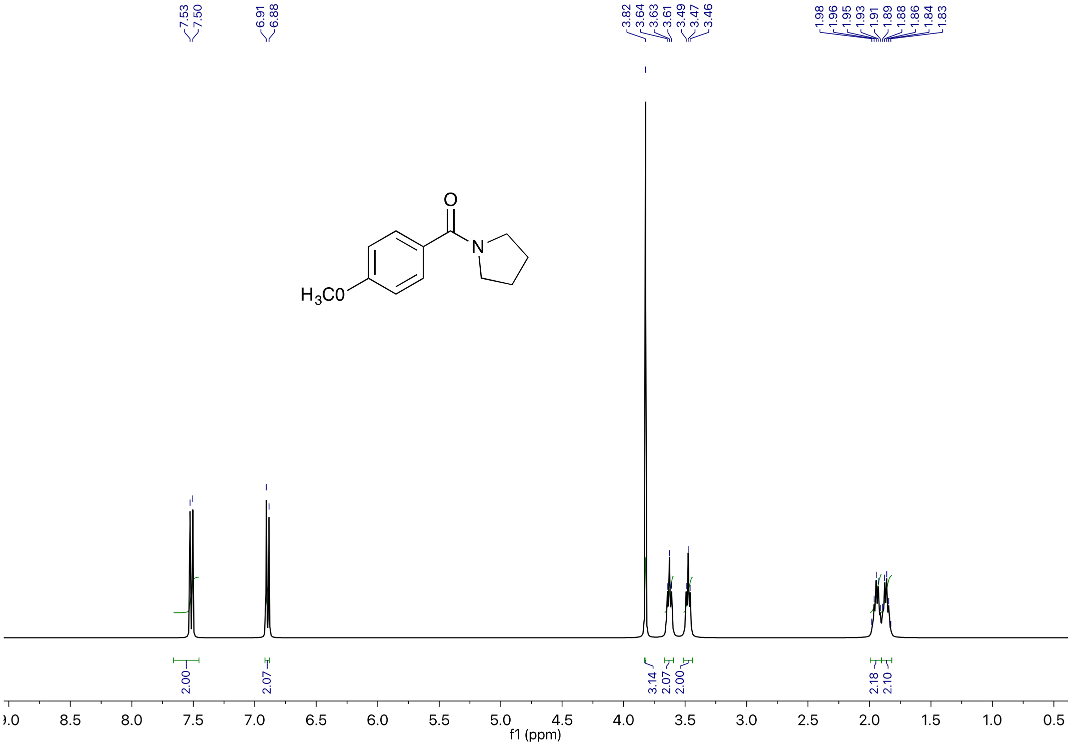

phenyl(pyrrolidin-1-yl)methanone (7c)
¹H NMR (400 MHz, $CDCl_3$)

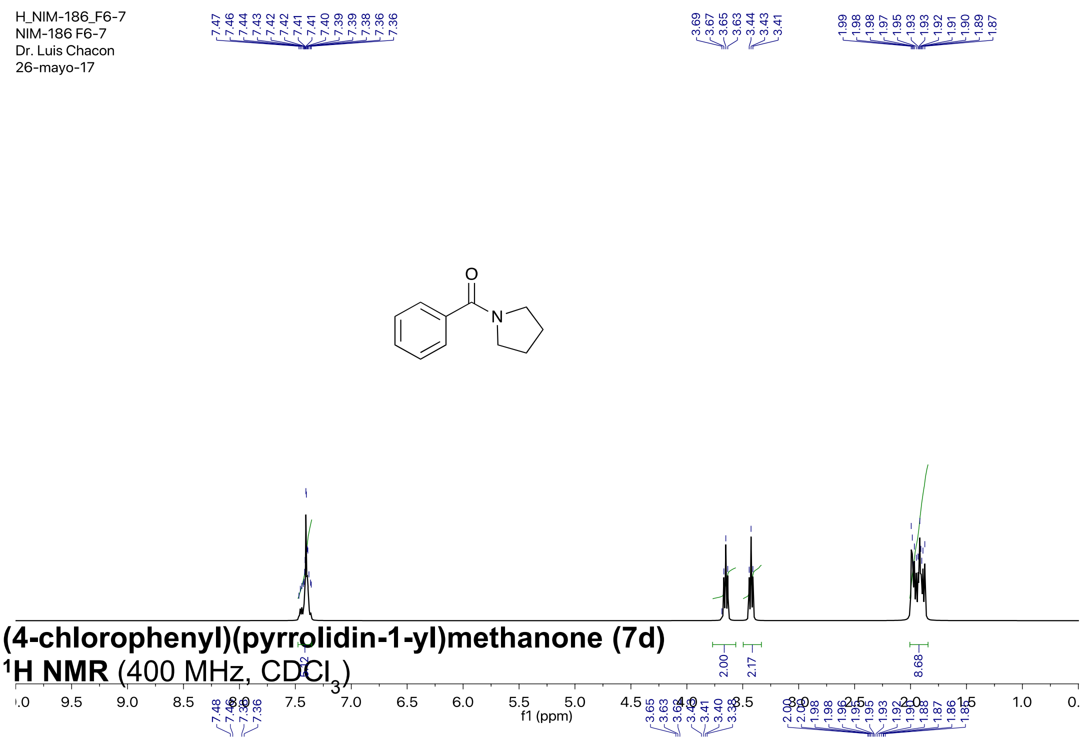

(4-chlorophenyl)(pyrrolidin-1-yl)methanone (7d)
¹H NMR (400 MHz, $CDCl_3$)

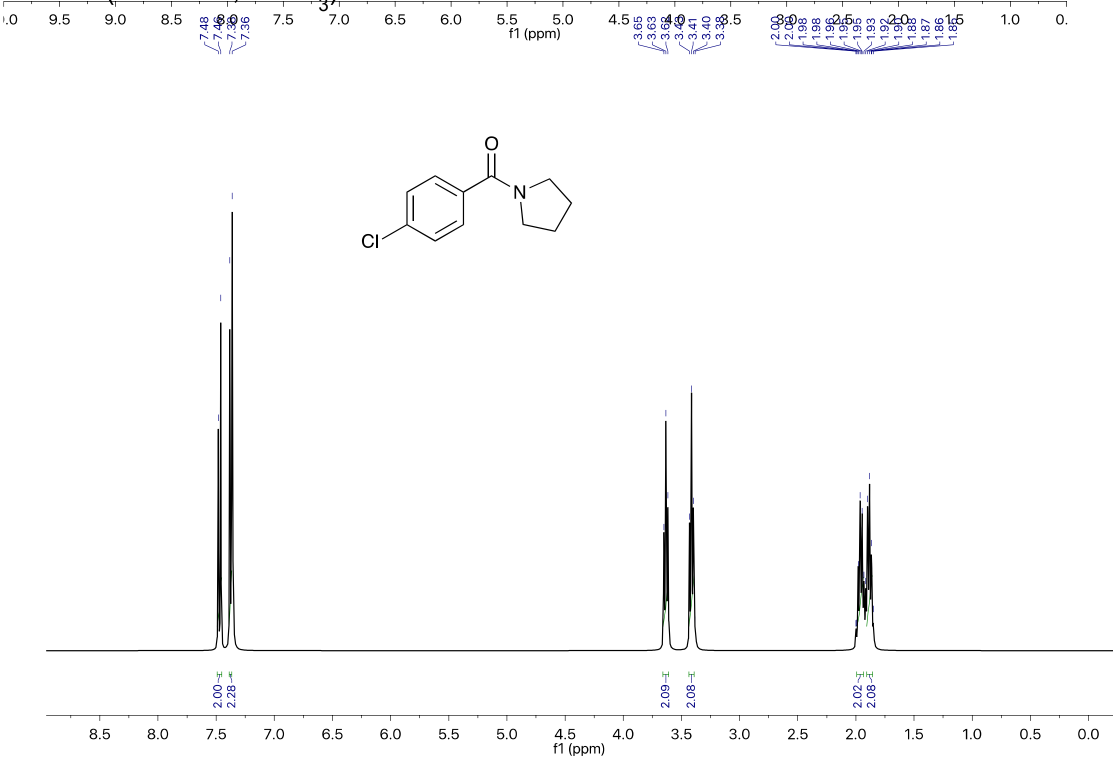

(4-bromophenyl)(pyrrolidin-1-yl)methanone (7e)
¹H NMR (400 MHz, $CDCl_3$)

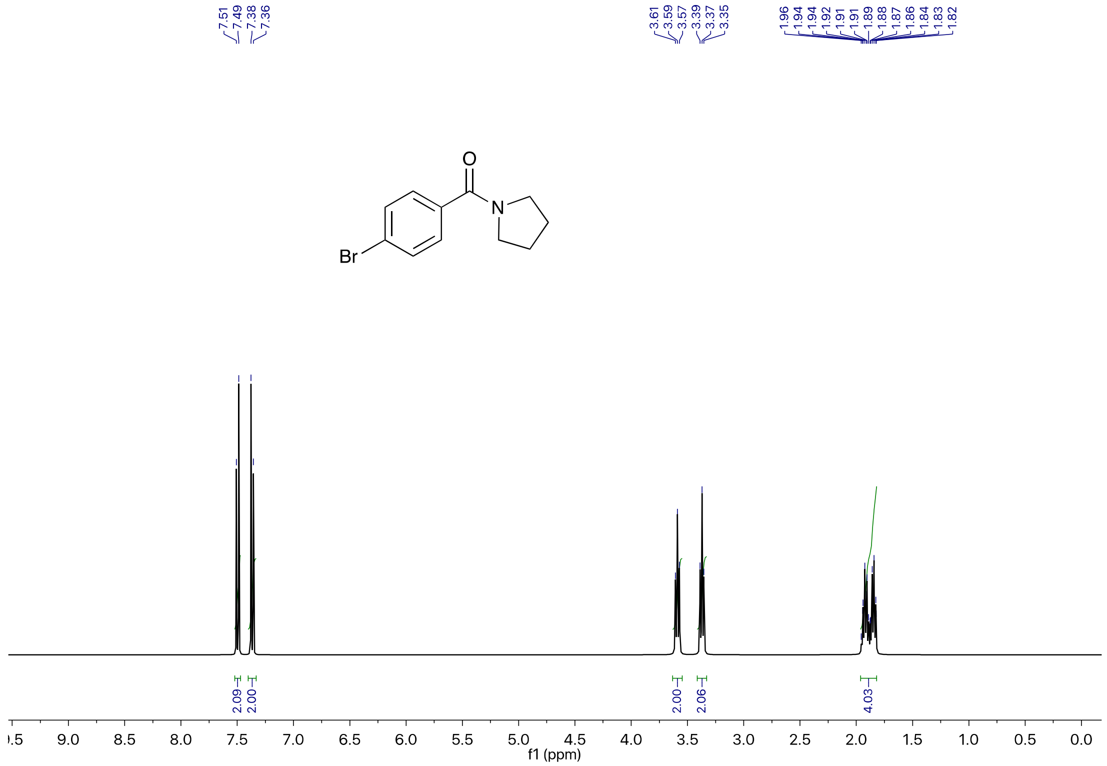

***N*,*N*-diethyl-4-nitrobenzamide (8)**

^{1}H NMR (400 MHz, $CDCl_3$)

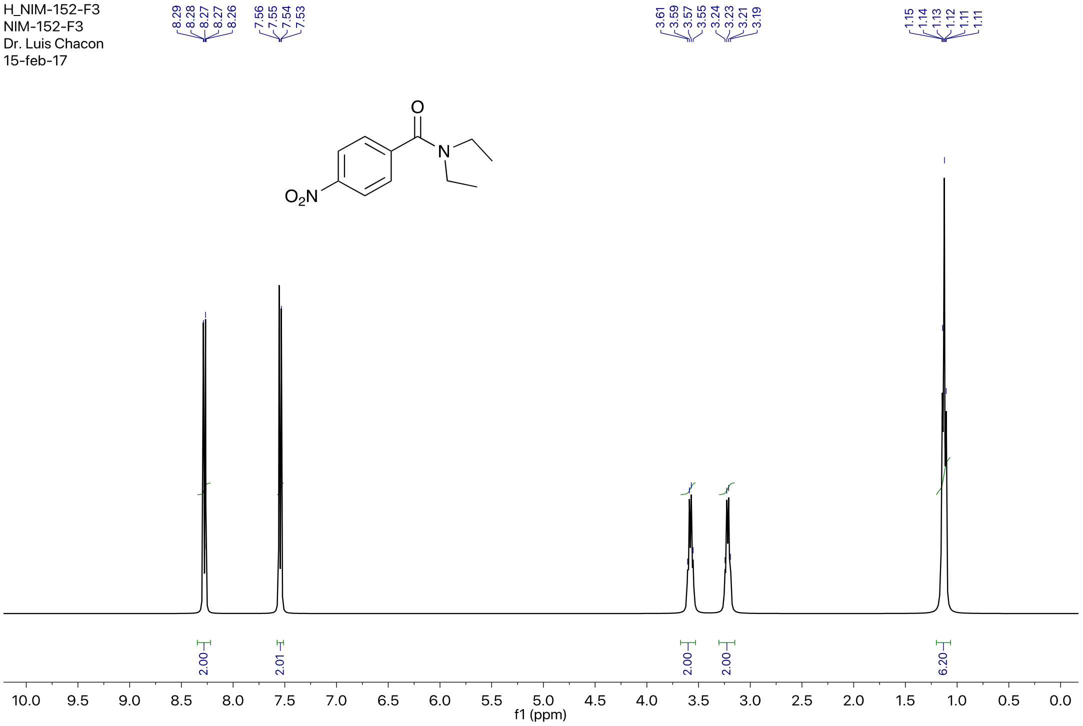

***N*,*N*-dibutyl-4-nitrobenzamide (8a)**

^{1}H NMR (400 MHz, $CDCl_3$)

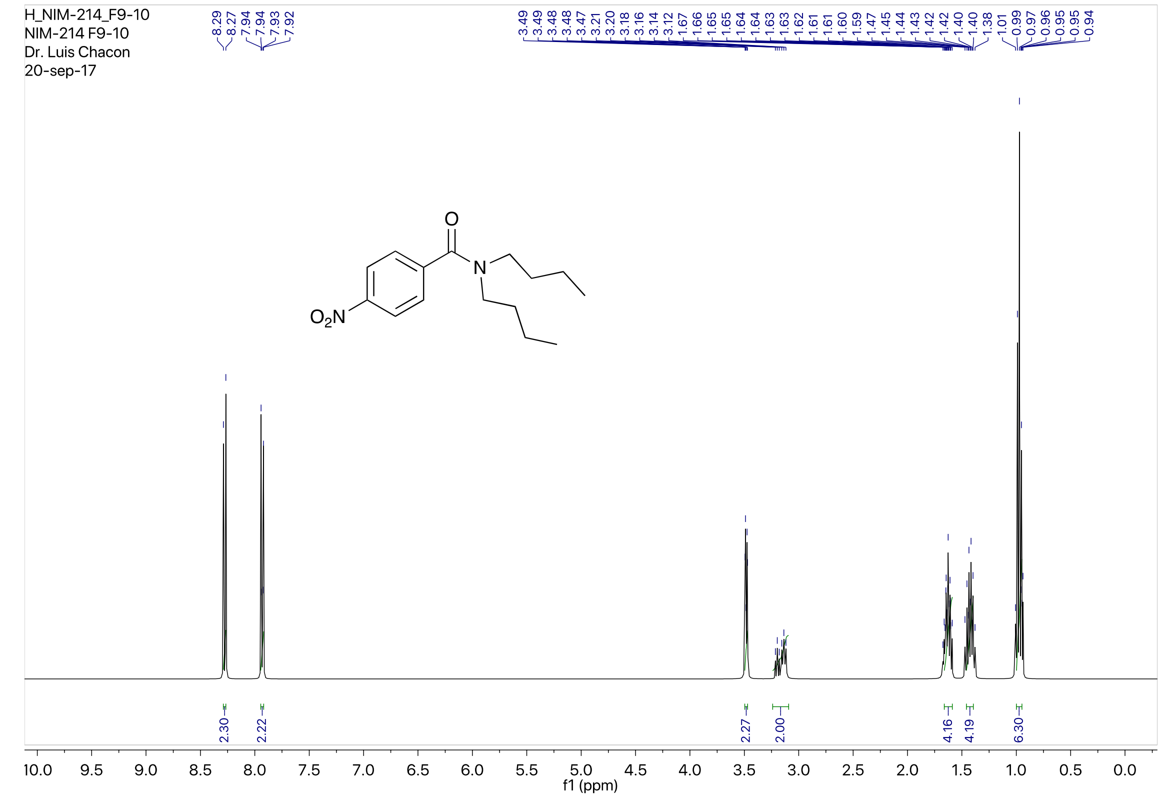

N-(4-Nitrobenzoyl)morpholine (8b)

^{1}H NMR (400 MHz, $CDCl_3$)

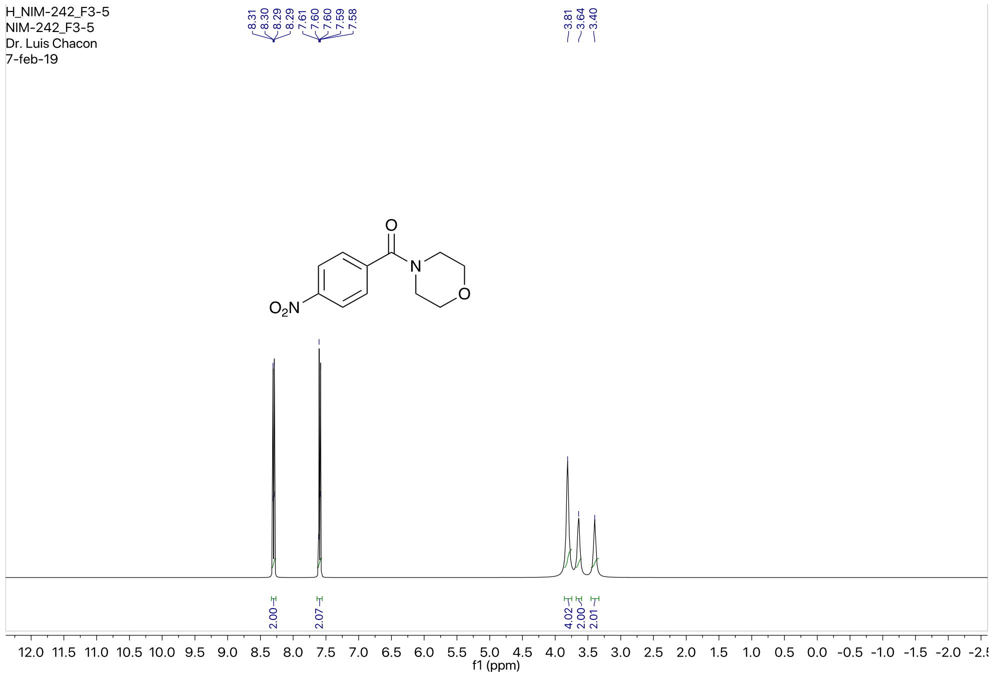

Piperazine-1,4-diylbis((4-nitrophenyl)methanone) (8c)
^{1}H NMR (400 MHz, $CDCl_3$)

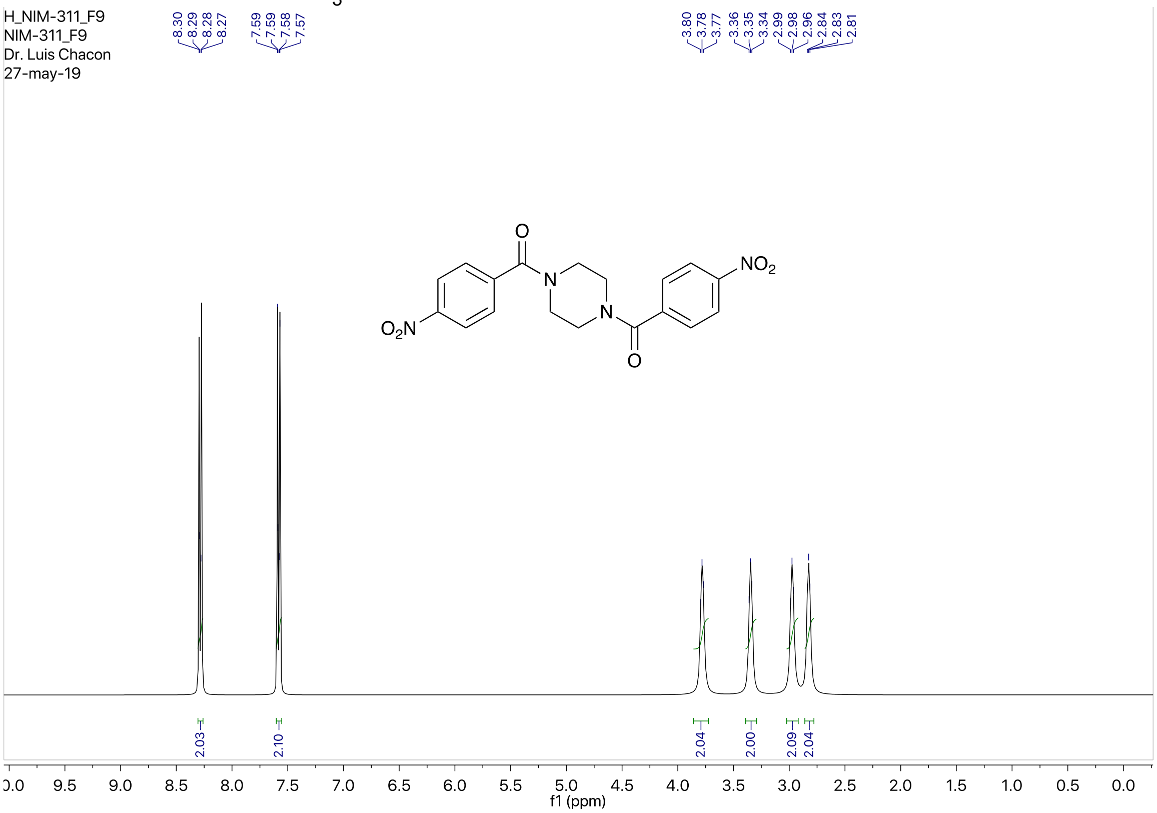

Theoretical calculations

Three definitions of $f(r)$ are obtained from the finite difference scheme,[6] which are helpful descriptors to evaluate a chemical specie for nucleophilic attacks $(f(r)^+)$, electrophilic attacks $(f(r)^-)$and for free radicals attacks $(f(r)^0)$ by using the following equations:

$$f(r)^+ = \rho_{N+1}(r) - \rho_N(r) \quad \textbf{(1)}$$
$$f(r)^- = \rho_N(r) - \rho_{N-1}(r) \quad \textbf{(2)}$$
$$f(r)^0 = 0.5(\rho_{N+1}(r) - \rho_{N-1}(r)) \quad \textbf{(3)}$$

where $\rho_{N+1}(r)$, $\rho_N(r)$ and $\rho_{N-1}(r)$ are the electronic densities at point r for the system with $N+1$, N and $N-1$electrons respectively.

The $f(r)^0$ form of the Fukui functions was used as a stability descriptor pursuing zones within the pyrrolyl quinones that could stabilize a free radical. The $f(r)^0$ descriptor indicated regions in the pyrrolyl quinones in which an unpaired electron could potentially be localized after redistribution of the initial electronic density.

The left panel of Scheme 1 presents the isocontours of $f(r)^0$ in the gas phase. The right side of Scheme 1 plots the isovalues of $f(r)^0$ in the presence of DMSO as the

solvent. The lowest energy conformers of **4** and its derivatives indicated the formation of a hydrogen bond between O_9 of the quinone and H of the pyrrole ring, with a length of 1.93, 1.92, 1.98, or 1.95 Å for **4**, **4a**, **4b**, and **4c**, respectively, in the gas phase. Although the presence of DMSO promoted hydrogen bonding, the hydrogen bond length increases with respect to the gas phase (being 1.98, 1.95, 2.01, and 1.99 Å for **4**, **4a**, **4b**, and **4c**, respectively).

The highest values of $f(r)^0$ suggested that the oxygen atoms O_8 and O_9 of the quinones were the most favorable sites for stabilizing a free radical, with a subtle preference for O_8 over O_9. O_9 participates in non-bonded interactions, whereas O_8 can accept one electron to form a radical. Radical formation raises an interesting question: Do the pyrrolyl quinones accept or donate the electron? To address this question, we calculated the values of $f(r)^+$ and $f(r)^-$ of the Fukui functions in the open shell scheme (after radical formation). The value $f(r)^+$ provides information about sites that stabilize incoming charges on the PQs. The value of $f(r)^-$ gives information about the electron donor sites from which a charge may "exit" to stabilize the PQs in a subsequent step.

Table 1 indicates that the highest values of the Fukui function occurred at O_8, particularly for $f(r)^+$. Once the radical formed, O_8 preferably accepted the incoming charge. It is important to note that $f(r)^+$ increased in the presence of DMSO by up to 7.2%, in agreement with our proposed mechanism that the quinones promoted radical formation in the presence of DMSO with synergic effects.

Table 1 Condensed forms of $f(r)^+$ and $f(r)^-$ (in e–), calculated after the radical formed in the PQs

Compound	Gas phase				DMSO			
	$f(r)^+$		$f(r)^-$		$f(r)^+$		$f(r)^-$	
	O_8	O_9	O_8	O_9	O_8	O_9	O_8	O_9
4	0.184	0.061	0.114	0.075	0.194	0.067	0.122	0.084
4a	0.181	0.069	0.129	0.078	0.194	0.120	0.066	0.083
4b	0.182	0.057	0.125	0.080	0.195	0.063	0.143	0.088
4c	0.184	0.061	0.120	0.078	0.196	0.067	0.138	0.087

**Compound**	**Gas Phase**	**DMSO**
**4**	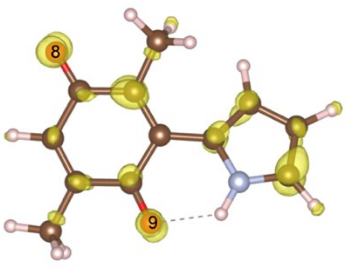	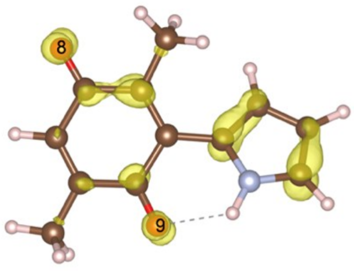
**4a**	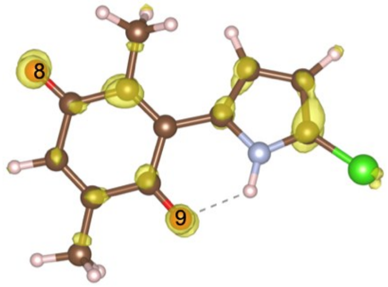	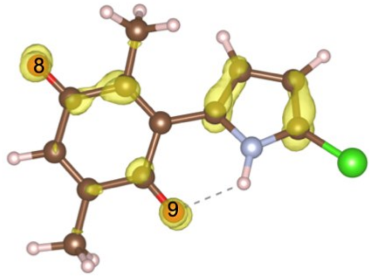

4b

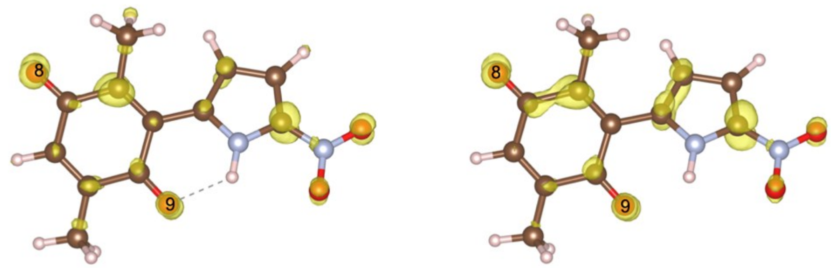

4c

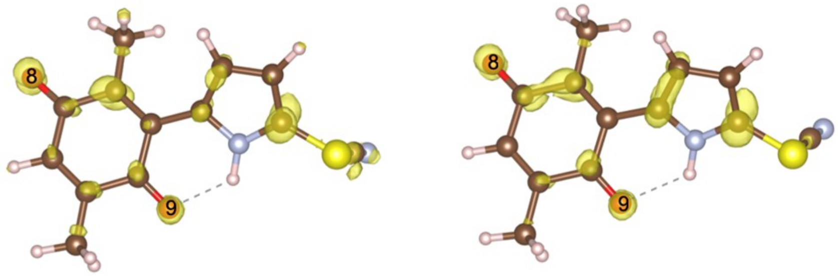

Scheme 1 Isocontours of f(r)0 for 4, 4a, 4b, 4c in the gas phase and in the presence of DMSO as a solvent

From the global properties of benzoquinones, we can clearly observe the solvent effect on such properties, e. g., electron affinity (**A**) increases approximately twice in the presence of the solvent with respect to the gas phase. In specific, the superiority of DMSO over CH_3CN can be observed analyzing the chemical hardness (**η**), which measures the resistance of a chemical specie to change in its electronic configuration[7] it decreases significantly in the presence of both CH_3CN and DMSO, finding in all cases, the lowest values of **η** in the presence of DMSO. Additionally, we observed that global electrophilicity (**ω**) of all species, increases considerably in the presence of the solvents, finding in the presence of DMSO the highest values of **ω**, which are up to 0.15 eV higher than **ω** calculated in the presence of CH_3CN. Other proofs of superiority of DMSO over CH_3CN (and other solvent with dissimilar polarity) we found analyzing the local reactivity descriptors, such as the condensed Fukui functions, which are part of a further publication. Tables 2, 3 and 4 list the results of the discussed global properties:

Table 2. Electron affinity (**A**), chemical potential (**μ**), hardness (**η**) and electrophilicity (**ω**) [in eV] of pyrrolyl benzoquinones calculated in the gas phase.

MOLECULE	***A***	***μ***	***η***	***ω***
NO_2	2.58	-5.04	4.91	2.58
SCN	2.36	-4.82	4.92	2.36
Cl	2.03	-4.46	4.87	2.05
H	1.87	-4.36	4.98	1.91

Table 3. Electron affinity (**A**), chemical potential (**μ**), hardness (**η**) and electrophilicity (**ω**) [in eV] of pyrrolil benzoquinones calculated in the presence of CH_3CN.

MOLECULE	*A*	*μ*	*η*	*ω*
NO_2	4.16	-4.96	1.61	7.65
SCN	3.92	-4.74	1.64	6.84
Cl	3.80	-4.57	1.53	6.81
H	3.73	-4.51	1.55	6.55

Table 4. Electron affinity (**A**), chemical potential (**μ**), hardness (**η**) and electrophilicity (**ω**) [in eV] of benzoquinones calculated in the presence of DMSO.

MOLECULE	*A*	*μ*	*η*	*ω*
NO_2	4.17	-4.96	1.58	7.78
SCN	3.93	-4.74	1.61	6.95
Cl	3.81	-4.57	1.51	6.92
H	3.75	-4.51	1.53	6.65

References

1. L. Chacon-García, M. Valle-Sánchez and C. Contreras-Celedon, *Lett. Org. Chem.*, 2013, **10**, 632.
2. É.-B. Hergovich and G. Speier, *J. Chem. Soc. Perkin Trans.,* 1986, **1**, 2305.
3. (a) H. R. Memarian, I. Mohammadpoor-Baltork and K. Nikoofar, *Ultrasonics Sonochemistry*, 2008, **15**, 456; (b) T. B. Mete, T. M. Khopade and R. G. Bhat, *Tetrahedron Lett.*, 2017, **58**, 415.
4. M. Tapia-Juárez, J. B. Gonzalez-Campos, C. Contreras-Celedón, D. Corona, E. Cuevas-Yañez and L. Chacon-García, *RSC Adv.,* 2014, **4**, 5660.
5. K.-E. Kovi and C. Wolf, *Org. Lett.*, 2007, **9**, 3429-3432.
6. P. W. Ayers, W. Yang, and L. J. Bartolotti, in Chemical Reactivity Theory: Chemical Reactivity Theory: A Density Functional View; Chattaraj, P.K., Ed.; CRC Press: Boca Raton, FL, USA, 2009
7. G. Macov, *J. Phys. Chem*. 1995, **99**, 9337.
